# *Lactiplantibacillus plantarum* FRT4 attenuates high-energy low-protein diet-induced fatty liver hemorrhage syndrome in laying hens through regulating gut-liver axis

**DOI:** 10.1186/s40104-023-00982-6

**Published:** 2024-02-21

**Authors:** Daojie Li, Hongying Cai, Guohua Liu, Yunsheng Han, Kai Qiu, Weiwei Liu, Kun Meng, Peilong Yang

**Affiliations:** 1 Key Laboratory of Feed Biotechnology of Ministry of Agriculture and Rural Affairs, Institute of Feed Research, Chinese Academy of Agricultural Sciences, Beijing, 100081 China; 2 National Engineering Research Center of Biological Feed, Beijing, 100081 China

**Keywords:** Fatty liver hemorrhage syndrome, Gut microbiota, *Lactiplantibacillus plantarum* FRT4, Laying hens, Lipid metabolism

## Abstract

**Background:**

Fatty liver hemorrhage syndrome (FLHS) becomes one of the most major factors resulting in the laying hen death for caged egg production. This study aimed to investigate the therapeutic effects of *Lactiplantibacillus plantarum* (*Lp. plantarum*) FRT4 on FLHS model in laying hen with a focus on liver lipid metabolism, and gut microbiota.

**Results:**

The FLHS model of laying hens was established by feeding a high-energy low-protein (HELP) diet, and the treatment groups were fed a HELP diet supplemented with differential proportions of *Lp. plantarum* FRT4. The results indicated that *Lp. plantarum* FRT4 increased laying rate, and reduced the liver lipid accumulation by regulating lipid metabolism (lipid synthesis and transport) and improving the gut microbiota composition. Moreover, *Lp. plantarum* FRT4 regulated the liver glycerophospholipid metabolism. Meanwhile, “gut-liver” axis analysis showed that there was a correlation between gut microbiota and lipid metabolites.

**Conclusions:**

The results indicated that *Lp. plantarum* FRT4 improved the laying performance and alleviated FLHS in HELP diet-induced laying hens through regulating “gut-liver” axis. Our findings reveal that glycerophospholipid metabolism could be the underlying mechanism for the anti-FLHS effect of *Lp. plantarum* FRT4 and for future use of *Lp. plantarum* FRT4 as an excellent additive for the prevention and mitigation of FLHS in laying hens.

**Supplementary Information:**

The online version contains supplementary material available at 10.1186/s40104-023-00982-6.

## Background

Non-alcoholic fatty liver disease (NAFLD) is one of the most central and affecting liver metabolic diseases worldwide, which is also defined as metabolic associated fatty liver disease (MAFLD) [1]. Generally, NAFLD is known as fatty liver hemorrhage syndrome (FLHS) in laying hens [2]. Genetics, nutrition, environment, and hormones are the main factors affecting the formation of FLHS, and lipid metabolism disorder and storage in liver are the major causes [3]. Actually, poultries require sufficient energy to maintain high laying performance in breeding industry. However, excess energy can be synthetized as lipids, which accumulate largely in liver and develop into FLHS in laying hens [4]. Nevertheless, cage systems remain by far the dominant way in egg production industry, with approximately 74% of caged laying hens dying from FLHS [5]. What’s worse, FLHS would lead to the decline of laying performance, causing huge economic losses [6].

As a functional additive with low cost and high safety, probiotics have emerged as an ideal strategy to attenuate the liver lipid metabolism disorder induced by the high energy diets [7]. Particularly, *Lactiplantibacillus plantarum* (*Lp*. *plantarum*) has been extensively employed in clinical settings for its safety and excellent efficacy. It has been reported that *Lp*. *plantarum* has a preventive effect on NAFLD [8]. What’s more, Loh et al. [9] reported that supplementation with 0.6% *Lp*. *plantarum* RI11, RG14 and RG11 metabolites decreased cholesterol level in plasma and yolk, and the content of cholesterol in eggs of Pengging duck was reduced by feeding *Lp. plantarum* Ina CC B76 mixed with inulin of gembili tuber [10]. There are few reports on the treatment of FLHS in laying hens by *Lp*. *plantarum*, and comprehensive studies on the lipid metabolism of *Lp. plantarum* in laying hens are scarce, and no relevant systematic study has been reported.

*Lp. plantarum* FRT4 was isolated, identified, and stored in our laboratory with CGMCC No.17955. Previous studies revealed that *Lp. plantarum* FRT4 regulated lipid metabolism and efficiently alleviated high-fat diet-induced obesity in mice through regulating gut microbiota [11, 12]. Therefore, we hypothesized that *Lp. plantarum* FRT4 had the efficiency to relieve FLHS in laying hens by regulating liver lipid metabolism and gut microbiota. This study was conducted to investigate the effect of *Lp. plantarum* FRT4 on liver lipid metabolism by liquid chromatography-mass spectrometry (LC-MS) and gas chromatography-mass spectrometry (GC-MS) analysis, and gut microbiota in laying hens with a high-energy low-protein (HELP) diet, which provided the evidences for the application of *Lp. plantarum* FRT4 to alleviate FLHS in laying hens.

## Materials and methods

### Ethics statement

Generated statement: The animal study was reviewed and approved by the Institutional Animal Care and Use Committee of the Institute of Feed Research of Chinese Academy of Agricultural Sciences (IFR-CAAS20220729).

### Experimental design and animal management

A total of 450 44-week-old Hy-line brown laying hens with the initial egg laying rate of 80.27% were randomly divided into 5 groups: normal diet (CT, control group), high-energy low-protein diet (HELP, model group), HELP diet supplemented with 10^9^ CFU/kg *Lp*. *plantarum* FRT4 (FRT4L group), HELP diet with 10^10^ CFU/kg *Lp*. *plantarum* FRT4 (FRT4M group), and HELP diet with 10^11^ CFU/kg *Lp*. *plantarum* FRT4 (FRT4H group). Each group contained 6 replications and each replication contained 15 hens. The laying hens were housed in a fully enclosed chicken house with a one-story ladder cage system and a relative humidity of appropriately 65% during the trial period and received water and diet ad libitum. After 2 weeks feeding normal diet, then the test was conducted and latest for 8 weeks. The laying rate, average daily feed intake (ADFI), and death rate were recorded daily. Average daily metabolic energy consumption (ADMEC) was calculated daily. Finally, the laying performance was corrected based on death rate.

The composition and nutrition contents of normal diet met the NRC (1994) [13], and the composition and nutrition contents of normal HELP diet referred to a previous study [14], which were shown in Additional file 1: Table S1.

### Sample collection

At the end of 8^th^ week, one laying hen per replicate was randomly selected for sampling after fasting for 12 h. The laying hens were euthanized via cutting the jugular vein for sample collection.

Liver and ovarian tissues were collected, and part of them were fixed in 4% neutral buffered paraformaldehyde for morphological observation and Oil Red O stain, and the other liver tissues were immediately frozen in liquid nitrogen, and then stored at −80 ºC. The caecal content was collected on ice and stored in liquid nitrogen immediately.

### Oil Red O staining of liver tissue

The fixed liver tissue was dehydrated and embedded by OCT embedding agent (G6059, Servicebio, Wuhan, China). The tissue was rough cut and sliced for 8 μm by a slicer (LEICA 819, LEICA, Shanghai, China). Then, the slices were stained with Oil Red O solution (G1015, Servicebio, Wuhan, China) in the dark, and covered with lid during dying. Next, the slices were immersed in 60% isopropanol for differentiation in turn (G1039, Servicebio, Wuhan, China). After that, the slices were stained by hematoxylin solution (G1004, Servicebio, Wuhan, China). Finally, sealing the slice with glycerin gelatin (G1402, Servivebio, Wuhan, China). The stain section was observed with microscope inspection (NIKON ECLIPSE E100, Nikon, Japan) and image system (NIKON DS-U3, Nikon, Japan).

### Biochemical assay of liver and ovarian tissues

The liver and ovarian tissues were washed with ice-cold phosphate buffered saline (PBS, pH 7.4), weighted 0.1 g and homogenized in 0.9 mL PBS or 1.0 mL isopropanol with a homogenizer. Then, the homogenates were centrifuged at 3,000 × *g* for 15 min at 4 ºC by a centrifuge (CT15RE, Hirachi Koki Co., Ltd., Tokyo, Japan). The supernatant was harvested for biochemistry parameters determination. The concentrations of triglyceride (TG), total cholesterol (TC), high-density lipoprotein cholesterol (HDL-C), low-density lipoprotein cholesterol (LDL-C), very low-density lipoprotein cholesterol (VLDL-C) in supernatant were measured with the corresponding kits (catalogue No. ml076637, ml076635, ml036973, ml036983, and ml060950, respectively) provided by Shanghai Enzyme-linked Biotechnology Co., Ltd. (Shanghai, China). All operations were carried out in accordance with the kits’ instructions. All the reading was performed with a microplate reader (1530, Thermo Fisher Scientific, Vantaa, Finland).

### qRT-PCR analysis

Total RNA was isolated from liver tissues by FastPure^®^ Cell/Tissue Total RNA Isolation Kit V2 (RC112-01, Vazyme Biotech Co., Ltd., Nanjing, China). RNA (0.1 µg) was used for reverse transcription by using Hiscript^®^ RT SuperMix for qPCR (+ gDNA wiper) kits (R323-01, Vazyme Biotech Co., Ltd., Nanjing, China). The reverse productions were used to perform qRT-PCR with the kits of Taq Pro Universal SYBR qPCR Master Mix (Q712-03, Vazyme Biotech Co., Ltd., Nanjing, China). The house keeping gene (*β-actin*) was assessed for the stability of expression. The expression level of each gene was calculated by using the 2^−ΔΔCt^ method. The primer sequences for all genes are presented in Additional file 1: Table S2.

### Untargeted liver metabolic analysis with LC-MS and GC-MS

#### Liver tissue metabolites extraction

The samples (30 mg) were accurately weighted into EP tubes (2 replications). Two small steel balls and 600 µL methanol (A452, Thermo Fisher Scientific, Waltham, MA, USA)-water (Wahaha, China) (v:v = 4:1, containing L-2-chlorophenylalanine (C2001, Shanghai Hengchuang Bio-technology Co., Ltd, Shanghai, China, 4 µg/mL)) were added into tubes. After pre-cooling in a refrigerator at −40 ºC for 2 min, the mixture was placed into a grinder (F-060SD, Fuyang Technology Co., Ltd., Shenzhen, China) for grinding (60 Hz, 2 min), and then ultrasonic extraction was put in an ice water bath for 10 min, and stewed at −40 ºC for 2 h (LC-MS) and 30 min (GC-MS). Subsequently, the mixed solution was centrifuged at 12,000 × *g* for 10 min at 4 ºC with a centrifuge (TGL-16MS, Luxiangyi Centrifuge Instrument Co., Ltd., Shanghai, China), and then the supernatant was filtered with a 0.22-μm organic phase pinhole filter. The filtrate was stored at −80 ºC for LC-MS analysis. Quality control (QC) samples were prepared by mixing aliquot of the all samples to be a pooled sample.

The sample using for GC-MS analysis was centrifuged at 12,000 × *g* for 10 min at 4 ºC. The supernatant (150 µL) was put into a glass derivative bottle and evaporated using a centrifugal concentration dryer. The methoxylamine hydrochloride solution (80 µL) (M813479, Macklin, Shanghai, China)-pyridine (P141169, Aladdin, Shanghai, China) was added into the glass derivative vial (15 mg/mL), and the mixed solution was placed in a shaking incubator (THZ-82, Lichen Bangxi Instrument Technology Co., Ltd., Shanghai) at 37 ºC for 60 min to undergo oximation reaction. BSTFA derivatization reagent (50 µL) (B0830, TCI, Japan), 20 µL N-hexane (4.011518.0500, CNW, Germany), and 10 µL per internal standard (C8/C9/C10/C12/C14/C16/C18/C20/C22/C24, configured with chloroform, G75915B, Greagent, Shanghai, China) were added into the reaction mixture. After reacting at 70 ºC for 60 min, the sample was kept at room temperature for 30 min for GC-MS metabolomic analysis. QC samples were prepared by mixing aliquot of the all samples to be a pooled sample.

#### LC-MS and GC-MS metabolomic analysis

LC-MS (Dionex U3000 UHPLC, QE plus, Thermo Fisher Scientific, Waltham, MA, USA) analysis conditions: chromatographic column (Acquity UPLC HSS T3, 100 mm × 2.1 mm × 1.8 μm, Waters, Milford, USA), and column temperature was 45 ºC. The mobile phases were A-water (containing 0.1% formic acid, A117-50, Thermo Fisher Scientific, Waltham, MA, USA), and B-acetonitrile (A998-4, Thermo Fisher Scientific, Waltham, MA, USA). The flow rate was 0.35 mL/min, and the injection volume was 5 µL. The UHPLC fitted with Q-Exactive Plus Quadrupole-Orbitrap mass spectrometer quipped with heated electrospray ionization (ESI) source (Thermo Fisher Scientific) was used to analyze the metabolic profiling in both ESI positive and ESI negative ion modes. The mass range was from 100 to 1,000 *m/z*. The resolution was set at 70,000 for the full MS scans and 17,500 for HCD MS/MS scans. The collision energy was set at 10, 20, and 40 eV. The mass spectrometer operated as follows: spray voltage, 3,800 V (+) and 3,000 V (−); sheath gas flow rate, 35 arbitrary units; auxiliary gas flow rate, 8 arbitrary units; capillary temperature, 320 ºC; Aux gas heater temperature, 350 ºC; S-lens RF level, 50. The QCs were injected at regular intervals throughout the analytical run to provide a set of data from which repeatability can be assessed.

GC-MS analysis conditions: DB-5MS capillary column (30 m × 0.25 mm × 0.25 μm, Agilent, Folsom, CA, USA). The carrier gas was high purity helium (purity not less than 99.999%). The flow rate was 1.0 mL/min, and the temperature of the sample inlet was 260 ºC. The injection volume was 1 µL. Without split injection, the solvent was delayed for 5 min. The programs of temperature rise: The initial temperature of the column temperature box was 60 ºC, maintained for 0.5 min, and then rose to 125 ºC at a rate of 8 ºC/min, to 210 ºC at a rate of 8 ºC/min, to 270 ºC at a rate of 15 ºC/min, to 305 ºC at a rate of 20 ºC/min and hold for 5 min. The temperature of MS quadrupole and ion source (electron impact) was set to 150 and 230 ºC, respectively. The collision energy was 70 eV. Mass spectrometric data was acquired in a full-scan mode (50–500 *m/z*). The QCs were injected at regular intervals throughout the analytical run to provide a set of data from which repeatability can be assessed.

### 16S rRNA sequencing and analysis

#### DNA extraction and amplification

Total DNA was extracted from the caecal contents using a DNeasy PowerSoil kit (Qiagen, Hilden, Germany) following the manufacturer’s instructions. DNA concentration and integrity were measured by a NanoDrop 2000 spectrophotometer (Thermo Fisher Scientific, Waltham, MA, USA) and agarose gel electrophoresis, respectively. PCR amplification of the V3-V4 hypervariable regions of the bacterial 16S rRNA gene was carried out in a 25 µL reaction system using universal primer pairs (343F: 5′-TACGGRAGGCAGCAG-3′, 798R: 5′-AGGGTATCTAATCCT-3′). The reverse primer contained a sample barcode and both primers were connected with an Illumina sequencing adapter.

#### Library construction and sequencing

The amplicon quality was visualized using gel electrophoresis. The PCR products were purified with Agencourt AMPure XP beads (Beckman Coulter Co., USA) and quantified using Qubit dsDNA assay kit. The concentrations were then adjusted for sequencing. Sequencing was performed on an Illumina NovaSeq 6000 with two paired-end read cycles of 250 bases each (Illumina Inc., San Diego, CA; OE Biotech Company, Shanghai, China).

#### Bioinformatic analysis

Raw sequencing data were in FASTQ format. Paired-end reads were then preprocessed using cutadapt software to detect and cut off the adapter. After trimming, paired-end reads were filtering low quality sequences, denoised, merged and detect and cut off the chimera reads using DADA2 with the default parameters of QIIME2. At last, the software output the representative reads and the amplicon sequence variants (ASVs) abundance table. The representative reads of each ASV were selected using QIIME2 package.

### Statistical analysis

The data were arranged using Excel (version 2019). The differences between two groups were analyzed by *t*-test of SPSS (version 25.0). The graphs were performed using GraphPad Prism version 8.0 (GraphPad Software, San Diego, CA, USA) and expressed as the mean ± SEM; *n* = 6 hens per group. *P* < 0.05 was considered to be significant difference.

The original LC-MS data were processed by software Progenesis QI (Version 2.3, Nonlinear, Dynamics, Newcastle, UK) for baseline filtering, peaking identification, integral, retention time correction, peak alignment, and normalization. The obtained GC-MS raw data were imported into software MS-DIAL, performing peak identification, MS2Dec deconvolution, characterization, peak alignment, wave filtering, and missing value interpolation. Metabolite characterization was based on LUG database. After the data were normalized, redundance removal and peak merging were conducted to obtain the data matrix. Orthogonal Partial Least-Squares-Discriminant Analysis (OPLS-DA) was utilized to distinguish the metabolites that differed between groups. To prevent overfitting, 7-fold cross-validation and 200 response permutation testing were used to evaluate the quality of the model. Variable importance of projection (VIP) values obtained from the OPLS-DA model were used to rank the overall contribution of each variable to group discrimination. A two-tailed Student’s *t*-test was further used to verify whether the metabolites of difference between groups were significant.

## Results

### Effect of *Lp. plantarum* FRT4 on laying performance of laying hens

**Fig. 1:**
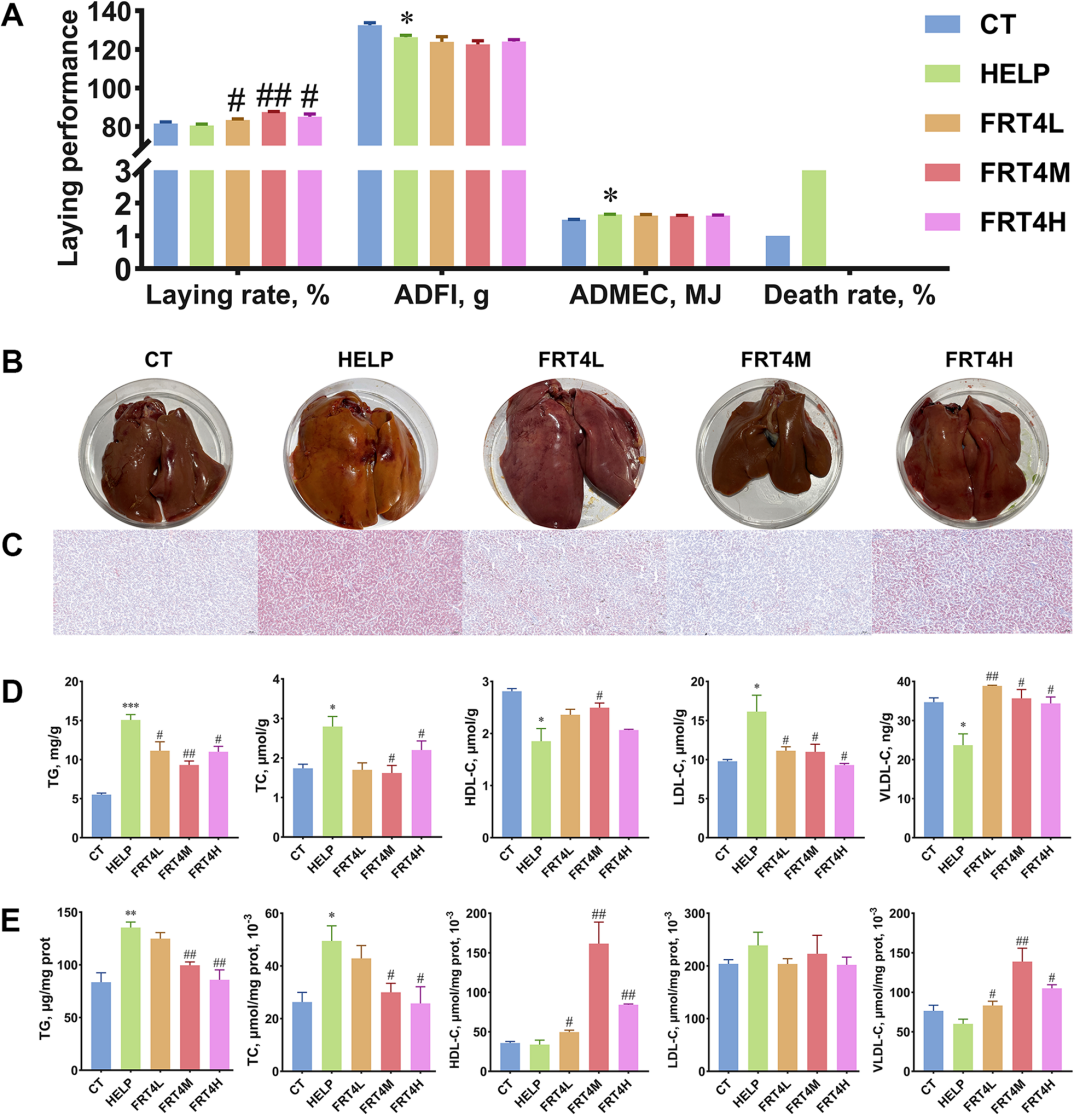
Effect of *Lp*. *plantarum* FRT4 on laying performance, liver morphology and biochemical parameters of laying hens. **A** The laying performance of laying hens in each group. The laying performance was corrected based on death rate. ADFI: Average daily feed intake; ADMEC: Average daily metabolic energy consumption. **B** Representative photographs of liver tissues of laying hens in each group. **C** Oil Red O staining observations of liver tissues of laying hens in each group (bar 100 μm). **D** Biochemical parameters of liver in each group. **E** Biochemical parameters of ovary in each group. The results were expressed as the mean ± SEM; *n* = 6 hens per group. * means the significant difference of HELP compared to CT group, and * means *P* < 0.05, ** means *P* < 0.01, *** means *P* < 0.001. # means the significant difference of FRT4L, FRT4M, and FRT4H compared to HELP group, and # means *P* < 0.05, ## means *P* < 0.01. CT: control group, hens fed with normal diet. HELP: model group, hens fed with high-energy low-protein diet. FRT4L, FRT4M, and FRT4H: experimental groups, hens fed with high-energy low-protein diet with 10^9^ CFU/kg, 10^10^ CFU/kg, and 10^11^ CFU/kg *Lp*. *plantarum* FRT4, respectively

During the period of test, the laying performance was recorded. As shown in Fig. 1A, the laying rate was decreased in the HELP group (80.63%) compared to the CT group (81.49%) (*P* > 0.05). After supplementation with *Lp. plantarum* FRT4, the laying rates was increased significantly in the FRT4L, FRT4M, and FRT4H groups (83.45%, 87.49%, and 85.11%, respectively) compared to the HELP group (*P* < 0.05). The ADFI was decreased significantly and ADMEC was increased in the HELP group compared to the CT group (*P* < 0.05). Compared to the HELP group, no significant difference was observed for the ADFI and ADMEC in *Lp. plantarum* FRT4 treatment groups (*P* > 0.05). In addition, the death rate in the CT group was 1%. However, the HELP group (3%) had higher death rate than the CT group. Importantly, *Lp. plantarum* FRT4 treatment groups had no death rate. Thus, *Lp. plantarum* FRT4 had the advantages on improving the laying performance of laying hens.

### Effect of *Lp. plantarum* FRT4 on liver and ovary biochemical indices of laying hens

A distinct dissimilarity of liver morphology was shown between CT group and HELP group (Fig. 1B). The liver was red in the CT group, while yellow in the HELP group, which was observed clearly. There was no significant difference between HELP and *Lp*. *plantarum* FRT4 intervention groups. Oil Red O stain of liver tissues showed that compared to the HELP group, the liver tissue in CT, FRT4L, FRT4M, and FRT4H groups contained lower lipid accumulation (Fig. 1C).

As shown in Fig. 1D, the contents of TC and TG in the HELP group were significantly higher than the CT group (*P* < 0.05), which were significantly reduced after *Lp*. *plantarum* FRT4 intervention. Furthermore, feeding HELP diet significantly reduced the contents of HDL-C and VLDL-C, and increased the content of LDL-C (*P* < 0.05) compared to the CT group, which were significantly reversed after *Lp*. *plantarum* FRT4 supplementation (*P* < 0.05).

As shown in Fig. 1E, compared to the CT group, the contents of TG and TC in HELP group were significantly increased (*P* < 0.05). No significant difference was observed for HDL-C, LDL-C, and VLDL-C between CT and HELP groups (*P* > 0.05). In the groups of FRT4M and FRT4H, the contents of TG and TC were significantly decreased after *Lp*. *plantarum* FRT4 supplementation (*P* < 0.05).

### Effect of *Lp. plantarum* FRT4 on lipid metabolism-related factors in liver

**Fig. 2:**
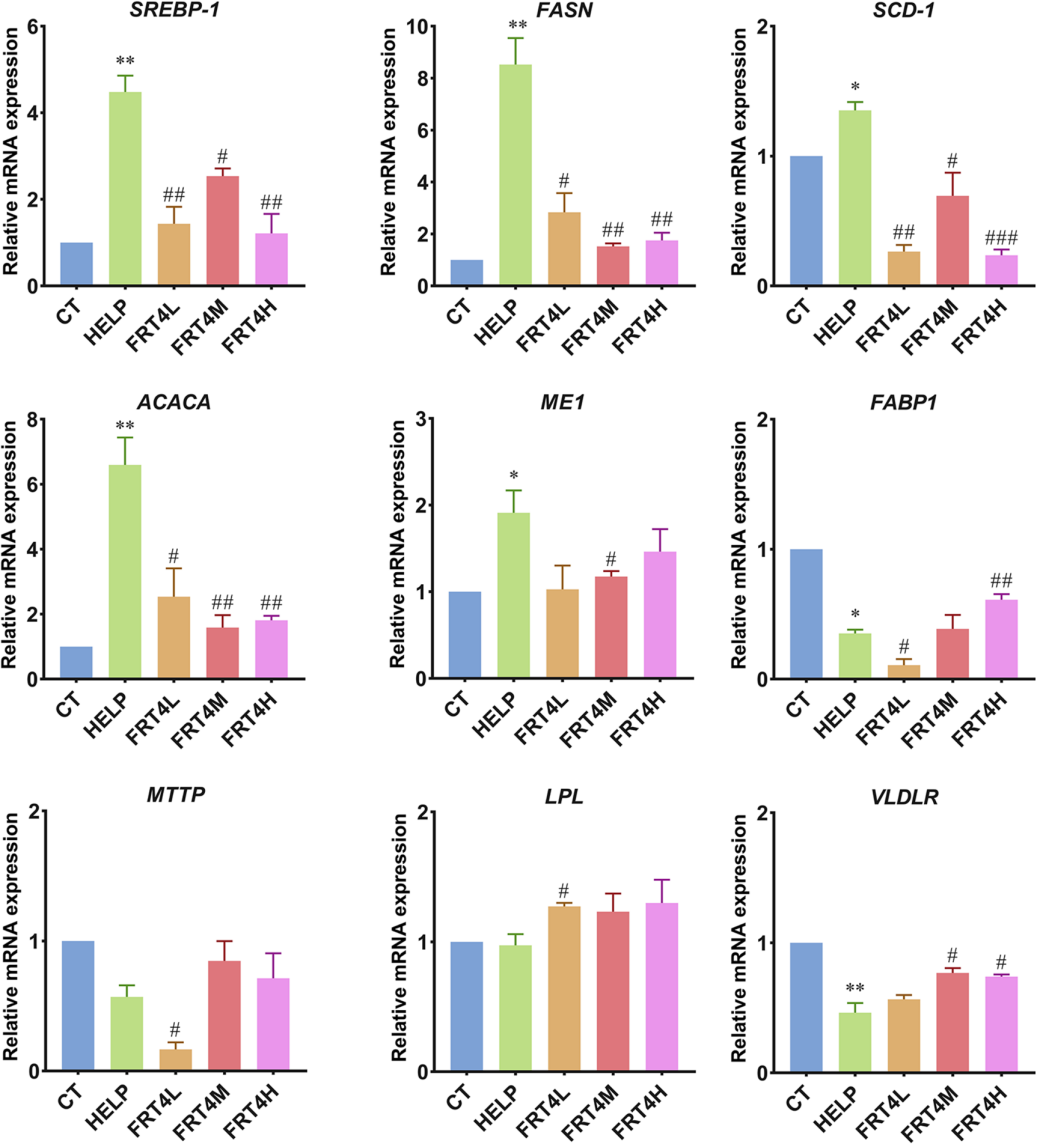
Effect of *Lp. plantarum* FRT4 on lipid metabolism-related factors in liver. The results were expressed as the mean ± SEM; *n* = 6 hens per group. * means the significant difference of HELP compared to CT group, and * means *P* < 0.05, ** means *P* < 0.01. # means the significant difference of FRT4L, FRT4M, and FRT4H compared to HELP group, and # means *P* < 0.05, ## means *P* < 0.01, ### means *P* < 0.001. CT: control group, hens fed with normal diet. HELP: model group, hens fed with high-energy low-protein diet. FRT4L, FRT4M, and FRT4H: experimental groups, hens fed with high-energy low-protein diet with 10^9^ CFU/kg, 10^10^ CFU/kg, and 10^11^ CFU/kg *Lp*. *plantarum* FRT4, respectively

The liver is the main organ of lipid homeostasis in the body, including lipid synthesis, transport, and β-oxidation. Compared to the CT group, the relative mRNA expressions of lipid synthesis including fatty acid synthase (*FASN*), stearoyl-CoA desaturase-1 (*SCD-1*), acetyl-CoA carboxylase alpha (*ACACA*), and malic enzyme 1 (*ME1*), and their upstream regulatory factor, sterol regulatory element binding protein 1 (*SREBP-1*), was significantly upregulated in the HELP group (*P* < 0.05) (Fig. 2), while was significantly downregulated after *Lp*. *plantarum* FRT4 supplementation (*P* < 0.05). The expression of fatty acid binding protein 1 (*FABP1*) and very low-density lipoprotein receptor (*VLDLR*), regulating liver lipid transport, was significantly downregulated (*P* < 0.05). The expression of *FABP1* in the FRT4H group and *VLDLR* in FRT4M and FRT4H groups was significantly upregulated (*P* < 0.05). The results indicated that *Lp. plantarum* FRT4 supplementation mainly regulated and improved the lipid synthesis and transport to reduce the hepatic lipid deposition.

### Effect of *Lp. plantarum* FRT4 on liver metabolites through untargeted metabonomic analysis

#### Multivariate statistical analysis of liver metabolites

**Fig. 3:**
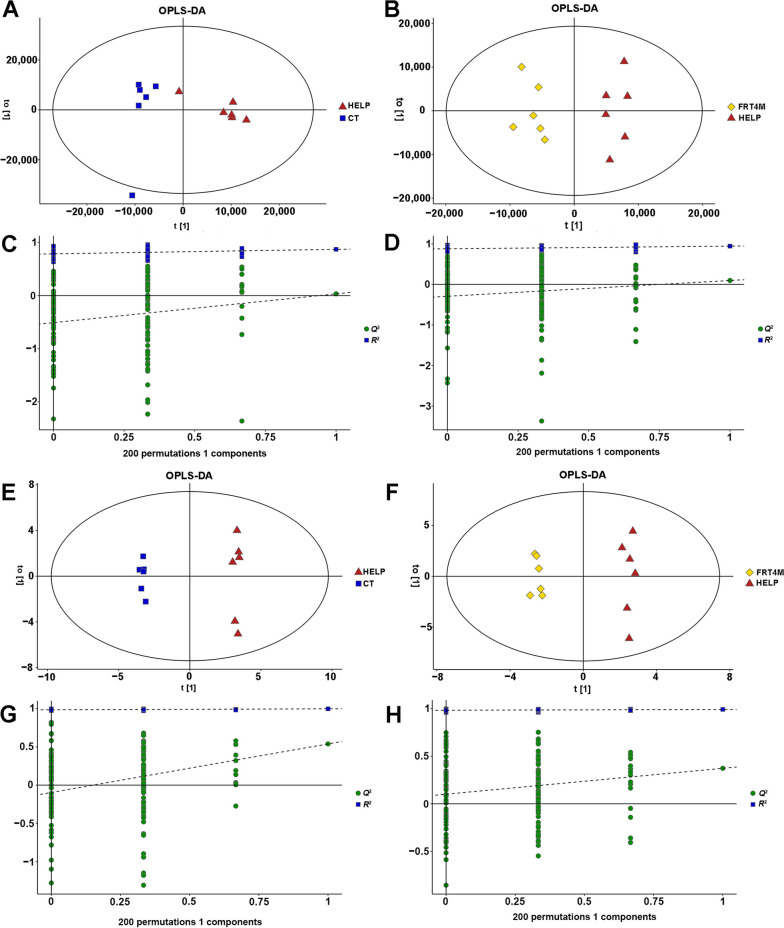
OPLS-DA score scatter plots of liver metabolites and permutation test displaying the stability of the OPLS-DA model. **A–D** LC-MS. **A** OPLS-DA score scatter of the HELP group compared to the CT group, *R*^2^*X* = 0.792, *R*^2^*Y* = 0.870, *Q*^2^ = 0.0324. **B** OPLS-DA score scatter of the FRT4M group compared to the HELP group, *R*^2^*X* = 0.618, *R*^2^*Y* = 0.944, *Q*^2^ = 0.0962. **C** permutation test displaying the stability of the HELP group compared to the CT group, *R*^2^ = 0.785, *Q*^2^ = −0.509. **D** the FRT4M group compared to the HELP group, *R*^2^ = 0.878, *Q*^2^ = −0.293. **E–H** GC-MS. **E** OPLS-DA score scatter of the HELP group compared to the CT group, *R*^2^*X* = 0.423, *R*^2^*Y* = 0.998, *Q*^2^ = 0.539. **F** OPLS-DA score scatter of the FRT4M group compared to the HELP group, *R*^2^*X* = 0.483, *R*^2^*Y* = 0.992, *Q*^2^ = 0.373. **G** permutation test displaying the stability of the HELP group compared to the CT group, *R*^2^ = 0.986, *Q*^2^ = −0.096. **H** the FRT4M group compared to the HELP group, *R*^2^ = 0.983, *Q*^2^ = 0.101. CT: control group, hens fed with normal diet. HELP: model group, hens fed with high-energy low-protein diet. FRT4M: experimental group, hens fed with high-energy low-protein diet with 10^10^ CFU/kg *Lp*. *plantarum* FRT4

After quality control (Additional file 2: Fig. S1–6), OPLS-DA was used to determine the metabolites, and permutation test displayed the stability of the OPLS-DA model. As shown in Fig. 3A and E, the OPLS-DA score scatter plot showed that there were significant differences between the HELP group and CT group, which indicated that HELP diet changed the liver metabolism of laying hens. Figure 3B and F showed that *Lp*. *plantarum* FRT4 intervention had significantly different clustering trends compared to the HELP group. Besides, the permutation test displayed the stability of the OPLS-DA model (Fig. 3C, D, G and H), indicating that OPLS-DA model had a good quality and was not over-fitted.

#### Differential metabolites analysis

Based on the OPLA-DA analysis, the VIP was used to measure the influence intensity and interpretation ability of the expression mode of each metabolite on the classification and discrimination. Further *t*-test was used to verify whether the different metabolites between two groups were significant. Finally, according to VIP > 1 and *P* < 0.05, 86 and 89 differential metabolites were identified for HELP compared to CT group and FRT4M compared to HELP group, respectively (see Additional file 3).

**Fig. 4:**
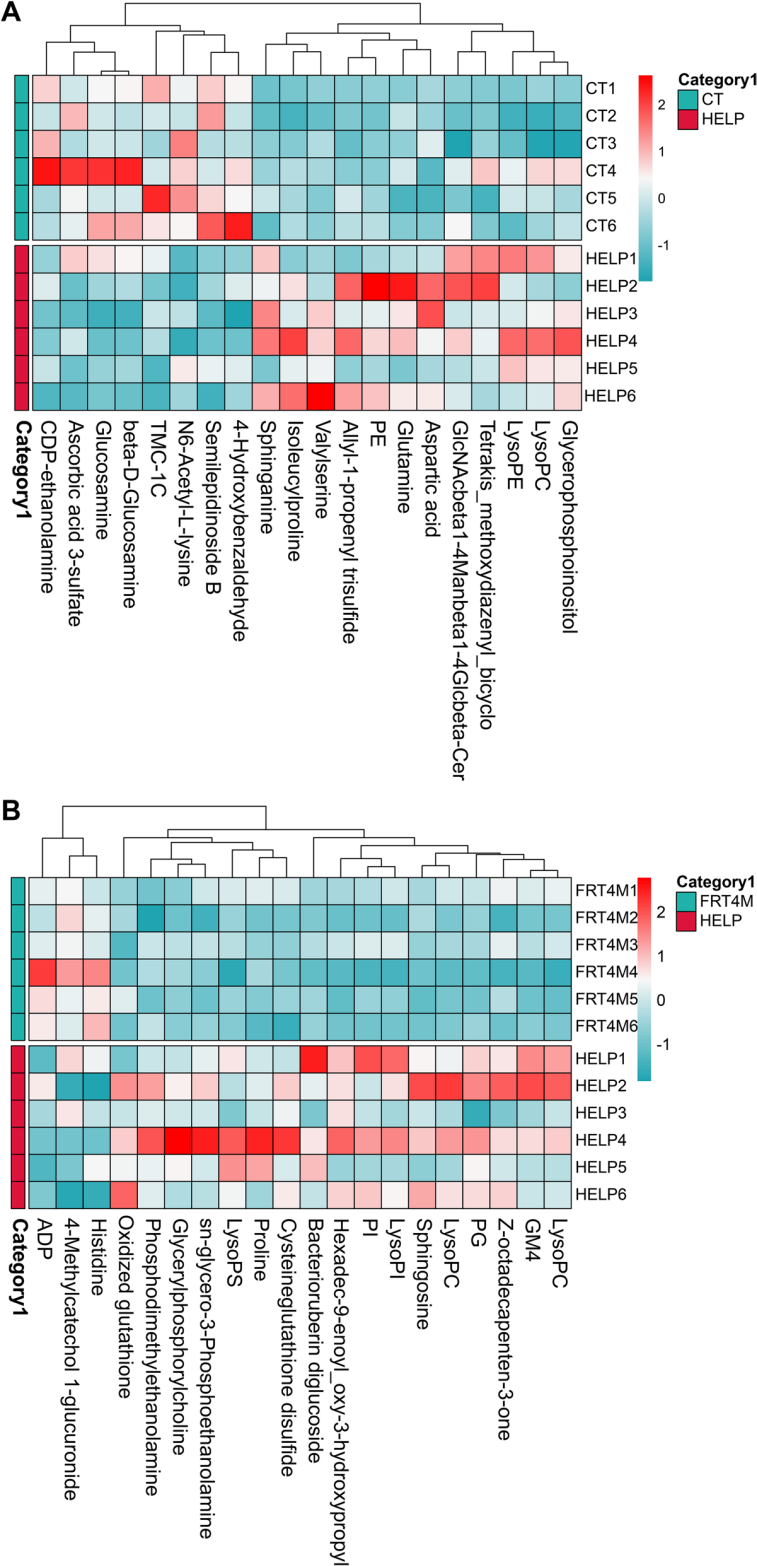
The heatmap plot of differential metabolites of laying hens’ liver. The abscissa represents the sample name, and the ordinate represents the difference metabolite. The color from blue to red indicates that the expression abundance of metabolites is from low to high, that is, the redder the expression abundance of differential metabolites is higher. **A** The heatmap plot of differential metabolites for HELP compared to CT group. **B** The heatmap plot of differential metabolites for FRT4M compared to HELP group. CT: control group, hens fed with normal diet. HELP: model group, hens fed with high-energy low-protein diet. FRT4M: experimental group, hens fed with high-energy low-protein diet with 10^10^ CFU/kg *Lp*. *plantarum* FRT4

Compared to the CT group, 19 differential metabolites involving carboxylic acids and derivatives were significantly altered in HELP group (Fig. 4A), including 9 increased metabolites like D-glutamine (*P* < 0.001), L-serine and alanylserine (*P* < 0.01), L-aspartic acid, isoleucylproline, valylserine, (2S)-2-amino-5-aminooxy-5-oxopentanoic acid, prolyl-lysine and (R)-propyl 2-amino-3-mercaptopropanoate (*P* < 0.05), and 10 decreased metabolites including L-2-aminobutanoic acid (*P* < 0.01), aminoadipic acid, beta-alanine, creatine, glycine, sarcosine, malonic acid, N6-acetyl-L-lysine, 5-aminopentanoic acid, N-linoleoyl methionine (*P* < 0.05). Ten potential biomarkers involving fatty acyls were significantly altered, including 3 increased metabolites like 3-hydroxyhexanedioylcarnitine (*P* < 0.001), *cis*-9-palmitoleic acid and oleic acid (*P* < 0.05), and 7 decreased metabolites including cetyl alcohol, alpha-linolenic acid, 4-hydroxybutyric acid, lauric acid, (E,E)-3,7,11-trimethyl-2,6,10-dodecatrienyl octanoate, TMC-1 C and 3-methylglutarylcarnitine (*P* < 0.05). Five differential metabolites involving glycerophospholipids were significantly increased, including LysoPC(16:0/0:0), LysoPE(22:5(7Z,10Z,13Z,16Z,19Z)/0:0), glycerophosphoinositol (*P* < 0.001), PE(O-18:0/0:0) and PIM1(19:1(9Z)/18:0) (*P* < 0.05). Twenty-six differential metabolites involving organonitrogen compounds were significantly altered, including 16 increased metabolites, such as sphinganine and N-acetyl-b-glucosaminylamine (*P* < 0.001), 1-butylamine, formycin B and aminofructose 6-phosphate (*P* < 0.01), sphingosine, D-gulose, beta-D-glucose 6-phosphate, and glucose 1-phosphate (*P* < 0.05), etc., and 10 decreased metabolites like gluconic acid, glucosamine, beta-D-glucosamine and semilepidinoside B (*P* < 0.001), shikimic acid (*P* < 0.01), putrescine, L-threonic acid, pantothenic acid, 5-(3´-hydroxyphenyl)-γ-valerolactone 3´-glucuronide and 4-hydroxybenzaldehyde (*P* < 0.05).

Compared to the HELP group, 18 potential biomarkers involving carboxylic acids and derivatives were significantly altered in FRT4M group (Fig. 4B), including 6 increased metabolites like L-histidine and prolyl-glutamine (*P* < 0.001), L-2-amino-3-(1-pyrazolyl) propanoic acid (*P* < 0.01), L-2-aminobutanoic acid, 3-methylhistidine and ophthalmic acid (*P* < 0.05), and 12 decreased metabolites like D-proline, gamma-glutamylglutamic acid (*P* < 0.001), N-acetylhistidine (*P* < 0.01), cysteineglutathione disulfide, oxidized glutathione, *N*-isobutyryl-L-cysteine and glycyl-prolyl-glutamic acid (*P* < 0.05), etc. Eight potential biomarkers involving fatty acyls were significantly altered. The metabolite cetyl alcohol was increased (*P* < 0.05), while 7 metabolites like docosahexaenoic acid, 2-hydroxydocosanoylcarnitine (*P* < 0.001), 2-hydroxycaproic acid (*P* < 0.01), aminocaproic acid, 2-hydroxyhexadecanal and 8-hydroxy-17-octadecene-10,12-diynoic acid (*P* < 0.05) were decreased. Twenty-four potential biomarkers involving glycerophospholipid metabolism were significantly decreased, including glycerylphosphorylcholine, glycerophosphocholine, LysoPC(18:0/0:0), LysoPC(20:1(11Z)/0:0), LysoPC(20:3(8Z,11Z,14Z)/0:0), LysoPC(20:2(11Z,14Z)/0:0), PC(17:1(9Z)/0:0), LysoPE(20:3(5Z,8Z,11Z)/0:0), LysoPE(20:1(11Z)/0:0), LysoPE(20:2(11Z,14Z)/0:0), LysoPE(16:1(9Z)/0:0) and LysoPE(0:0/20:1(11Z)) (*P* < 0.001), PG(18:3(6Z,9Z,12Z)/0:0) (*P* < 0.01), PC(22:1(11Z)/0:0), PC(O-16:0/3:1(2E)) and LysoPI(18:1(9Z)/0:0) (*P* < 0.05), etc. Eleven potential biomarkers involving organonitrogen compounds were significantly altered, including 3 increased metabolites like gluconic acid (*P* < 0.01), 4-methylcatechol 1-glucuronide 5-(3´-hydroxyphenyl)-γ-valerolactone 3´-glucuronide (*P* < 0.05), and 8 decreased metabolites like sphingosine, (2R,3R)-2-aminooctadecane-1,3-diol and 4-hydroxysphinganine (*P* < 0.001), melibiose and aminofructose 6-phosphate (*P* < 0.01), glucose 1-phosphate, maltotriose and 3h-adrenaline (*P* < 0.05).

#### Differential metabolic pathway analysis

**Fig. 5:**
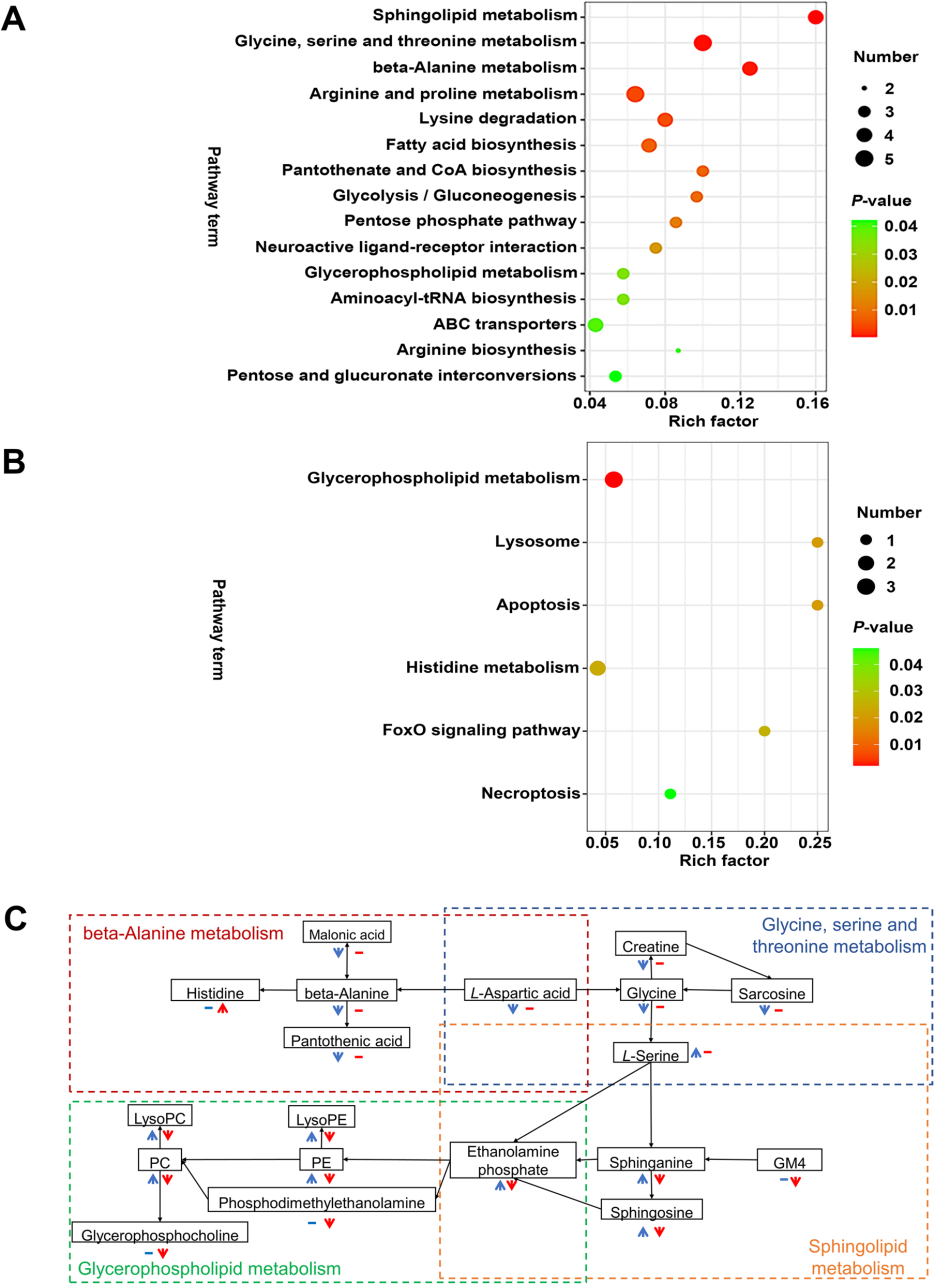
The bubble plot differential metabolic pathway analysis based on differential metabolites. The ordinate is the name of metabolic pathway. The abscissa is the enrichment factor (Rich factor = number of significantly different metabolites/total number of metabolites in the path). The larger the Rich factor, the greater the enrichment degree. The color from green to red indicates that *P*-value decreases in turn. The larger the point, the more metabolites enriched on the pathway. **A** The bubble plot differential metabolic pathway analysis based on differential metabolites for HELP compared to CT group. **B** The bubble plot differential metabolic pathway analysis based on differential metabolites for FRT4M compared to HELP group, respectively. The ordinate is the name of metabolic pathway. The abscissa is the enrichment factor (Rich factor). The larger the Rich factor, the greater the enrichment degree. The color from green to red indicates that *P*-value decreases in turn. The larger the point, the more metabolites enriched on the pathway. **C** Interactive metabolic pathway map based on the KEGG database involving glycerophospholipid metabolism, sphingolipid metabolism, glycine, serine and threonine metabolism, beta-Alanine metabolism. Under the metabolites, blue represents HELP compared to CT group, red represents FRT4M compared to HELP group. The up arrow means the metabolite was significant upregulation, and the down arrow means the metabolite was significant downregulation. Horizontal line means no significant difference between groups. CT: control group, hens fed with normal diet. HELP: model group, hens fed with high-energy low-protein diet. FRT4M: experimental group, hens fed with high-energy low-protein diet with 10^10^ CFU/kg *Lp*. *plantarum* FRT4

The differential metabolites were analyzed through pathway enrichment analysis based on Kyoto Encyclopedia of Genes and Genomes (KEGG) database. There were 50 and 28 metabolic pathways identified in HELP group compared to CT group and FRT4M group compared to HELP group, respectively (see Additional file 4). Furthermore, metabolic pathway was considered significantly differential pathway according to *P* < 0.05. Fifteen metabolic pathways were significantly different in HELP compared to the CT group (Fig. 5A), including sphingolipid metabolism, glycine, serine and threonine metabolism, beta-alanine metabolism, arginine and proline metabolism, lysine degradation, fatty acid biosynthesis, pantothenate and CoA biosynthesis, glycolysis/gluconeogenesis, pentose phosphate pathway, neuroactive ligand-receptor interaction, and glycerophospholipid metabolism, etc. Six metabolic pathways were significantly different in FRT4M group compared to the HELP group (Fig. 5B), including glycerophospholipid metabolism, lysosome, apoptosis, histidine metabolism, foxO signaling pathway, and necroptosis. It’s worth noting that the glycerophospholipid metabolism was the most significantly different pathway. Besides, the interactive metabolic pathway map based on the KEGG database involving glycerophospholipid metabolism, sphingolipid metabolism, glycine, serine and threonine metabolism, and beta-alanine metabolism was shown in Fig. 5C.

### Effect of *Lp. plantarum* FRT4 on glycerophospholipid metabolism-related factors in liver

**Fig. 6:**
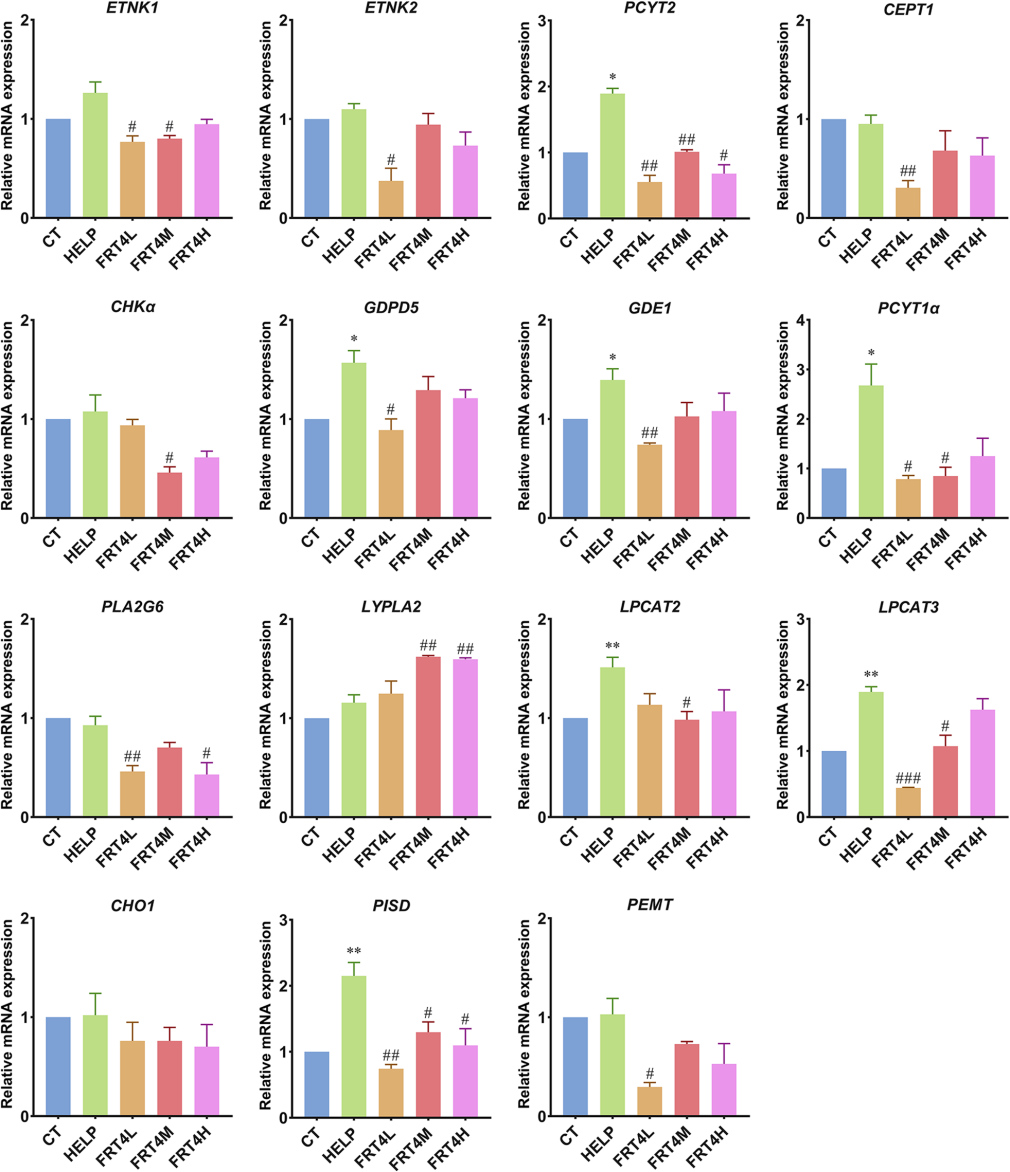
Effect of *Lp. plantarum* FRT4 on glycerophospholipid metabolism-related factors in liver. The results were expressed as the mean ± SEM; *n* = 6 hens per group. * means the significant difference of HELP compared to CT group, and * means *P* < 0.05, ** means *P* < 0.01. # means the significant difference of FRT4L, FRT4M, and FRT4H compared to HELP group, and # means *P* < 0.05, ## means *P* < 0.01, ### means *P* < 0.001. CT: control group, hens fed with normal diet. HELP: model group, hens fed with high-energy low-protein diet. FRT4L, FRT4M, and FRT4H: experimental groups, hens fed with high-energy low-protein diet with 10^9^ CFU/kg, 10^10^ CFU/kg, and 10^11^ CFU/kg *Lp*. *plantarum* FRT4, respectively

According to the metabolomic analysis, we found that glycerophospholipid metabolic pathway played an important role in alleviated FLHS in laying hens intervened by *Lp. plantarum* FRT4. Thus, we investigated the related genes expression involving in glycerophospholipid metabolic pathway in liver (Fig. 6). The key gene expression of phosphate cytidylyltransferase 1 alpha (*PCYT1*α), phosphate cytidylyltransferase 2 (*PCYT*2), glycerophosphodiester phosphodiesterase 1 (*GDE*1), glycerophosphodiester phosphodiesterase domain containing 5 (*GDPD*5), lysophosphatidylcholine acyltransferase 2 (*LPCAT*2), *LPCAT*3, and phosphatidylserine decarboxylase (*PISD*) in glycerophospholipid metabolic pathway were significantly increased in HELP group compared to the CT group (*P* < 0.05). The *PCYT*2 and *PISD* expression levels intervened by *Lp. plantarum* FRT4 were significantly decreased compared to the HELP group (*P* < 0.05).

### Effect of *Lp. plantarum* FRT4 on caecal content microbiota of laying hens

**Fig. 7:**
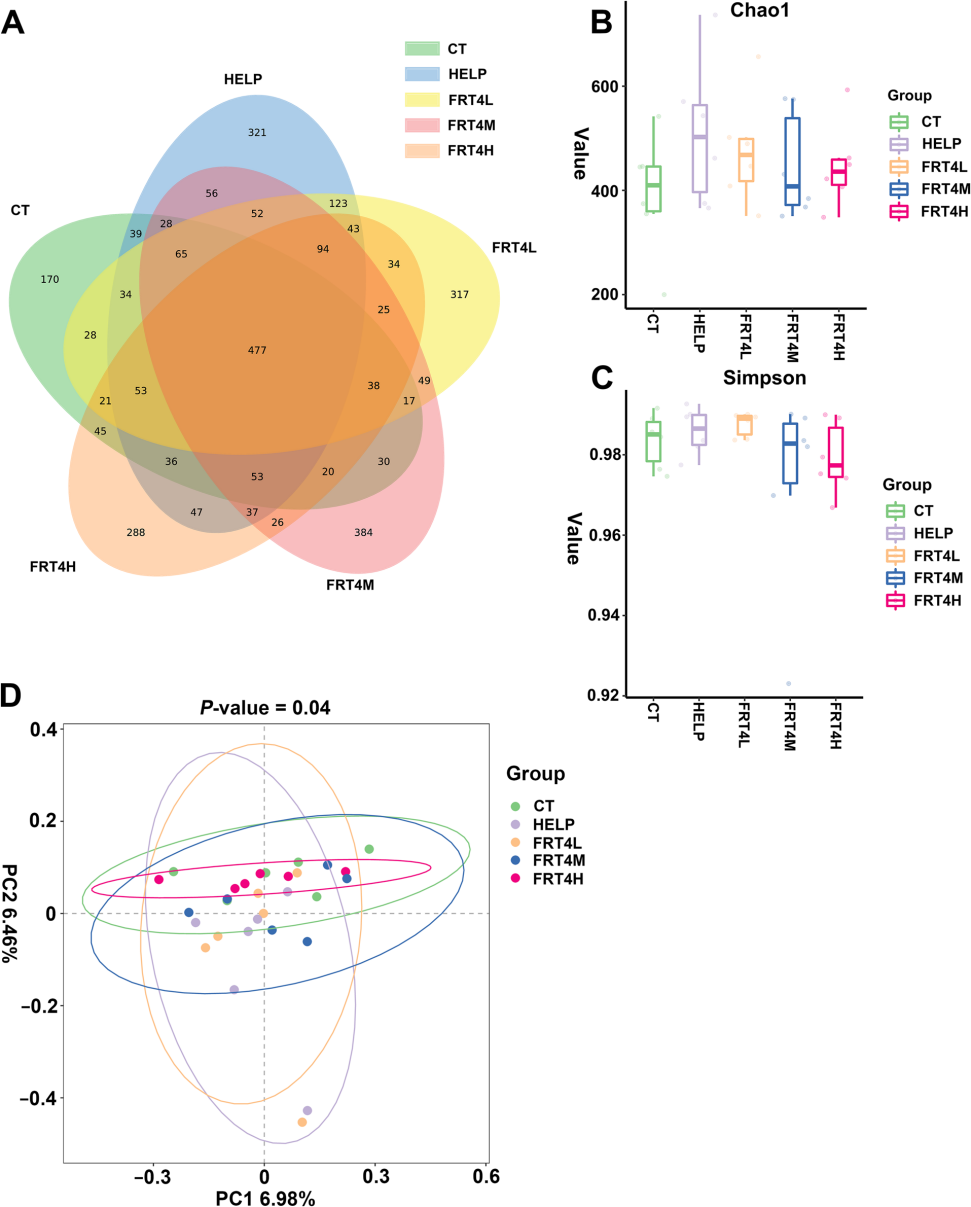
Effect of *Lp*. *plantarum* FRT4 on gut microbiome diversity indices. **A** Venn plot of gut microbiome ASVs number of each group. **B** The Chao1 index of α-diversity. **C** The Simpson index of α-diversity. **D** PCoA analysis with 95% confidence interval by different color for each group (*P* = 0.04). CT: control group, hens fed with normal diet. HELP: model group, hens fed with high-energy low-protein diet. FRT4L, FRT4M, and FRT4H: experimental groups, hens fed with high-energy low-protein diet with 10^9^ CFU/kg, 10^10^ CFU/kg, and 10^11^ CFU/kg *Lp*. *plantarum* FRT4, respectively

A total of 3,030 ASVs were observed in this study. 1,154 ASVs with 170 unique ASVs, 1,158 ASVs with 321 unique ASVs, 1,470 ASVs with 317 unique ASVs, 1,451 ASVs with unique 384 ASVs, and 1337 ASVs with 288 unique ASVs were identified in CT, HELP, FRT4L, FRT4M, and FRT4H groups, respectively (Fig. 7A). Although the difference was not significant, the Chao1 and Simpson indices of α-diversity increased in HELP, which was reserved after *Lp. plantarum* FRT4 supplementation (Fig. 7B and C). PCoA analysis showed that the microbiota community structure in HELP group (purple interval) and FRT4L group (orange interval) differed from the CT group (shown with green interval) (Fig. 7D). The community structures of FRT4M (blue interval) and FRT4H (red interval) groups were more similar to the CT group.

**Fig. 8:**
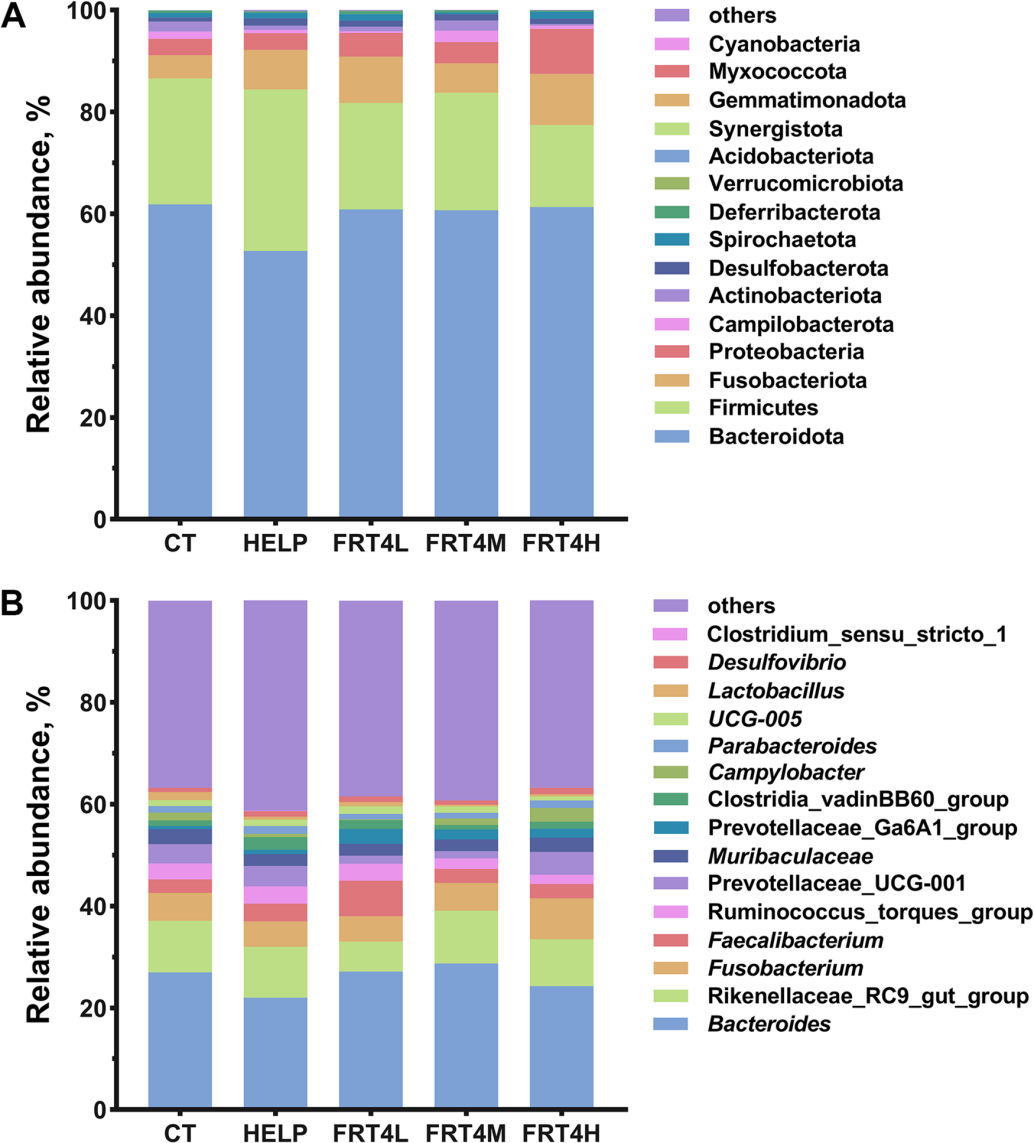
Effect of *Lp*. *plantarum* FRT4 on gut microbiome composition. **A** Microorganisms with the top 15 relative abundances at phylum level. **B** Microorganisms with the top 15 relative abundances at genus level. CT: control group, hens fed with normal diet. HELP: model group, hens fed with high-energy low-protein diet. FRT4L, FRT4M, and FRT4H: experimental groups, hens fed with high-energy low-protein diet with 10^9^ CFU/kg, 10^10^ CFU/kg, and 10^11^ CFU/kg *Lp*. *plantarum* FRT4, respectively

The top 15 relative abundance microorganisms of the microbiota composition at different levels were calculated. At phylum level (Fig. 8A), Bacteroidota, Firmicutes, Fusobacteriota, Proteobacteria, and Campilobacterota were the dominant phyla in all groups, and total relative abundance of which were more than 95%. The laying hens fed HELP diet reduced the relative abundance of Bacteroidota, Fusobacteriota, Proteobacteria and Campilobacterota, and the ratio of Firmicutes to Bacteroidota (F/B), while supplied with *Lp*. *plantarum* FRT4 reconstructed these microbes. At the genus level, *Bacteroides*, *Rikenellaceae*_RC9_gut_group, *Fusobacterium*, *Prevotellaceae*_UCG-001, *Muribaculaceae*, [*Ruminococcus*]_torques_group, *Campylobacter*, and *Lactobacillus* were the dominant genera in all groups (Fig. 8B). The relative abundances of *Fusobacterium*, *Prevotellaceae*_UCG-001, *Muribaculaceae*, *Campylobacter*, *Lactobacillus* were decreased, and the relative abundances of *Bacteroides*, *Faecalibacterium*, and *Prevotellaceae*_Ga6A1_group were increased in HELP group compared to the CT group, while *Lp*. *plantarum* FRT4 supplementation reconstructed these microbes as well.

**Fig. 9:**
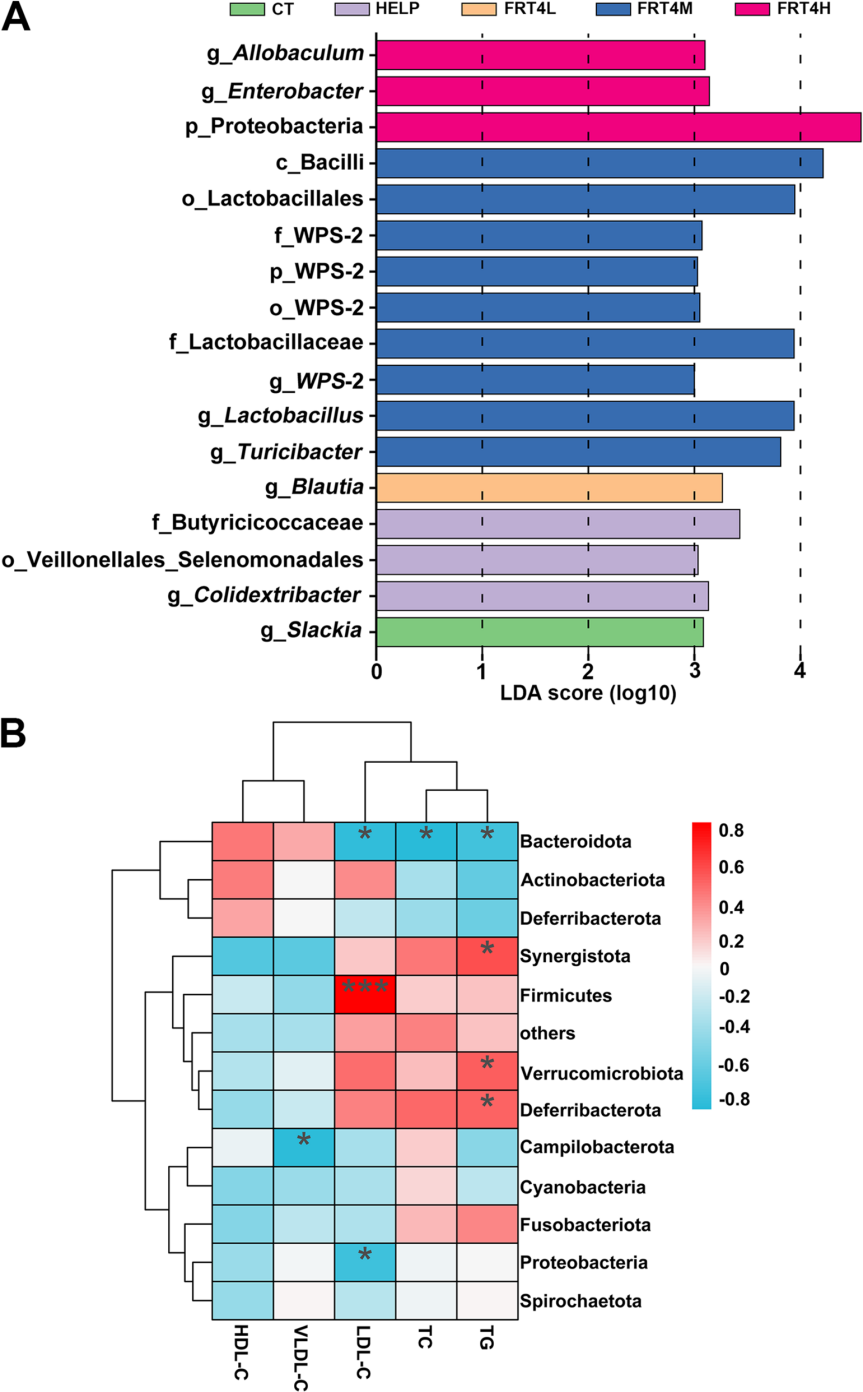
Effect of *Lp*. *plantarum* FRT4 on biomarkers and correlation with liver lipid biochemical parameters of gut microbiome. **A** LEfSe analysis of differential species for biomarkers on microbiota structure in groups. Different color expresses different group. The abscissa is the LDA score (log10), and the ordinate is the species in different categorical level. **B** The correlation heatmap between gut microbiota and liver lipid biochemical parameters. The red means positive correlation, and the blue means negative correlation. * means *P* < 0.05, *** means *P* < 0.001. CT: control group, hens fed with normal diet. HELP: model group, hens fed with high-energy low-protein diet. FRT4L, FRT4M, and FRT4H: experimental groups, hens fed with high-energy low-protein diet with 10^9^ CFU/kg, 10^10^ CFU/kg, and 10^11^ CFU/kg *Lp*. *plantarum* FRT4, respectively

The linear discriminant analysis (LDA) effect size (LEfSe) analysis was used to identify the significant difference between groups (LDA > 3) using LEfSe software. In the CT group, *Slackia* was the biomarker (Fig. 9A). In the HELP group, Butyricicoccaceae and *Colidextribacter* were the biomarkers. Blautia was the biomarker in FRT4L group. Bacilli, Lactobacillales, *Lactobacillus*, WPS-2, and *WPS*-2 were the biomarkers in FRT4M group. Proteobacteria, *Enterobacter*, and *Allobaculum* were the biomarkers in FRT4H group.

*Lp. plantarum* FRT4 intervention not only altered the composition of gut microbiota, but also improved the liver lipid metabolism. Therefore, the correlation between gut microbiota and liver lipid parameters was analyzed by Spearman correlation analysis (Fig. 9B). The results showed that Bacteroidota was negatively related with TG, TC, and LDL-C (*P* < 0.05). Synergistota, Desulfbacterota, and Verrucomicrobiota were positively related with TG (*P* < 0.05). Firmicutes was positively related with LDL-C (*P* < 0.001), while Proteobacteria was negatively with LDL-C (*P* < 0.05). The correlated results suggested that there was interaction between liver lipids and gut microbiota, which demonstrated the beneficial roles of *Lp. plantarum* FRT4 on the regulation of gut microbiota in FLHS of laying hens.

## Discussion

FLHS is one of the most serious liver metabolic diseases that causes decrease in the laying rate and unexpected death of laying hens, which has brought great losses to the breeding industry. Although FLHS has been explored for a long time, there are no efficient methods to solve the problem. In this study, *Lp. plantarum* FRT4 was used to treat the FLHS in laying hens induced by HELP diet, aiming to clarify the regulatory mechanisms that *Lp. plantarum* FRT4 alleviated the FLHS in laying hens.

In recent years, HELP diet has become the popular approach to construct FLHS chicken model [14, 15]. The TG accumulation in liver was often considered the main characteristic of FLHS [16]. The hens suffering FLHS or liver lipid metabolism disorder would have lower laying production [17, 18]. In our study, the laying rate was decreased after feeding HELP diet, and the concentrations of TG, TC, and LDL-C in HELP group were higher than the CT group. Meanwhile, the representative photographs and Oil Red O stain of liver tissues showed that the liver steatosis occurred in HELP group. These results indicated that the FLHS model in laying hens was successfully established, which was consistent with the reported reference [14]. However, supplementation with *Lp. plantarum* FRT4 significantly increased the laying rate, and reduced the concentrations of TG, TC, and LDL-C in liver. The concentrations of HDL-C and VLDL-C in HELP were significantly lower than the CT group. Meanwhile, supplementation with *Lp. plantarum* FRT4 significantly increased the concentrations of HDL-C and VLDL-C in liver compared to the HELP group, which did not differ from the CT group. Jia et al. [19] considered the density of lipoprotein was related to the transport of cholesterol as well as HDL-C and VLDL-C has been considered to have a protective effect for liver lipid metabolism, while LDL-C was harmful to liver. Batista et al. [20] considered high level of VLDL could promote lipolysis. Importantly, our previous study suggested that *Lp. plantarum* FRT4 reduced the TG and GLU contents in liver and serum in HFD-induced obese mice [12]. Evidences above suggested that *Lp. plantarum* FRT4 could reduce the lipid deposition induced by HELP diet in laying hens.

*SREBP-1*, *FASN*, *SCD-1*, *ACACA*, and *ME1* are the key genes involved in lipid synthesis [21]. *SREBP-1* is the important transcription factor regulating the lipid biosynthesis and uptake. FSAN is the key enzymes that catalyzes fatty acid synthesis. ACACA is the important fatty acid rate-limiting enzyme. SCD-1 and ME1 are the major enzymes that catalyze lipogenesis. HELP diet significantly upregulated the expressions of *SREBP-1*, *FASN*, *SCD-1*, *ACACA*, and *ME1* in liver of laying hens, resulting in lipid accumulating. However, supplementation with *Lp. plantarum* FRT4 reversed the expression increase of these genes, and reduced the lipid synthesis in liver. FABP is related with lipid transport [22]. VLDLR, LPL, and microsomal triglyceride transfer protein (MTTP) usually play a crucial role in transport and metabolism of lipoprotein and lipid [23, 24]. The expressions of *FABP1* and *VLDLR* were significantly decreased by HELP diet, while were upregulated after *Lp. plantarum* FRT4 intervention. Therefore, *Lp. plantarum* FRT4 intervention reduced the lipid accumulation in laying hens through reducing lipid synthesis and promoting lipid transport.

In order to clarify the role of *Lp. plantarum* FRT4 in adjusting liver metabolism in FLHS, untargeted metabolic assay was used through LC-MS and GC-MS analysis. Previous studies reported that sphingolipid was an important biomarker for NAFLD [25]. Concurrently, researchers have proclaimed that glycerophospholipid metabolism was one of the most important pathways in influencing liver lipid metabolism [26]. In the present study, sphingolipid metabolism associated with sphingolipids metabolites, such as L-serine, sphingosine, sphinganine and o-phosphoethanolamine, were significantly upregulated in HELP group compared to the CT group, as well as glycerophospholipid metabolism associated with glycerophospholipid metabolites like PE(O-18:0/0:0), LysoPC(16:0/0:0), LysoPE(22:5(7Z,10Z,13Z,16Z,19Z)/0:0) and glycerophosphoinositol were significantly upregulated. Sphingolipids are bioactive lipids regulating organism biology and could be found in livers of NAFLD [27]. Therefore, HELP diet could cause the FLHS in laying hens through gathering lipid in liver. Compared to the HELP group, glycerophospholipid metabolism associated with 23 glycerophospholipid metabolites, such as LysoPC(18:0/0:0), LysoPC(20:1(11Z)/0:0), glycerylphosphorylcholine, glycerophosphocholine, PC(17:1(9Z)/0:0), LysoPC(20:3(8Z,11Z,14Z)/0:0), LysoPE(16:1(9Z)/0:0), etc., were significantly downregulated in FRT4M group. Phosphatidylcholine (PC) and phosphatidylethanolamine (PE) were the main parts of glycerophospholipids [26]. Vinaixa et al. [28] considered that animals, suffering from NAFLD, had a higher conversion of PC and PE in liver. Meanwhile, glycerophospholipids and sphingolipids were the important metabolites affecting the development and progression of NAFLD [29]. In the present study, the concentrations of PC and PE were significantly increased in the HELP group, while decreased significantly in the FRT4M group. Therefore, it has compelling reasons to convince that *Lp. plantarum* FRT4 ameliorated FLHS in laying hens by the way of improving glycerophospholipid metabolism.

The results of liver metabonomic analysis indicated the glycerophospholipid metabolic pathway (gga00564) played a crucial role in the occurrence and development of FLHS. Herein, we determined the correlated genes expression level of glycerophospholipid metabolism to clarify the regulatory mechanism of alleviating FLHS by *Lp. plantarum* FRT4. PC is one of the most abundant phospholipids [30], as well as phospholipids are esters of glycerol, fatty acids, phosphoric acid, and other alcohols. PE is the substrate of lipid peroxidation [31]. PCYT1α [EC: 2.7.7.15] and PCYT2 [EC: 2.7.7.14] were reported importantly associated with PC and PE de novo synthesis [32, 33]. The expression levels of *PCYT1*α and *PCYT*2 were significantly increased resulting from HELP diet, while significantly decreased after *Lp. plantarum* FRT4 intervention. The expressions of key genes *PISD* [EC: 4.1.1.65] and ethanolamine kinase (*ETNK*, [EC: 2.7.1.82]) had the similar expression trends, catalyzing the PS decarboxylation for synthesis of PE [34]. Furthermore, the liver metabonomic results showed that the contents of glycerophospholipid metabolites were significantly decreased in liver after *Lp. plantarum* FRT4 treatment. A previous study reported that glycerophospholipids played a vital role in the development of NAFLD [35]. Concurrently, other related genes of PE and PC metabolism (M00090, M00091, M00092, M00093), such as choline/ethanolamine phosphotransferase 1 (*CEPT1* [EC: 2.7.8.1]), choline kinase alpha (*CHKα* [EC: 2.7.1.32]), *GDPD5* [EC: 3.1.4.2], phospholipase A2 group VI (*PLA2G6* [EC: 3.1.1.4]), *LYPLA2* [EC: 3.1.1.5], *LPCAT2* and *LPCAT3* [EC: 2.3.1.23], were regulated by *Lp. plantarum* FRT4. The results indicated that *Lp. plantarum* FRT4 treatment decreased the expression of glycerophospholipid metabolism-related factors to reduce the glycerophospholipid metabolites synthesis, enhance the glycerophospholipid metabolites metabolism, and protect laying hens against FLHS.

Substantial studies have supported that the gut microbiota plays a pivotal role in NAFLD. The gut microbiota composition always keeps a relatively stable state of laying hens, and the dominant phyla were Bacteroidota, Firmicutes, Proteobacteria, Actinobacteriota, and Fusobacteriota. The higher of Firmicutes and lower Bacteroidota, as well as descended F/B, were the characteristic of NAFLD [36]. Alferink et al. [37] perceived that hepatic steatosis was usually associated with *Ruminococcus*. Higher abundance of Firmicutes and Ruminococcaceae, and lower Bacteroidota was found in the HELP group, while *Lp. plantarum* FRT4 supplementation decreased the abundances of Firmicutes and Ruminococcaceae, and increased the abundance of Bacteroidota. In addition, the correlation analysis indicated that TG, TC, and LDL-C showed a negative correlation with Bacterioda, and LDL-C was positive correlation with Firmicutes. Wang et al. [38] reported that *Clostridium butyricum* could affect lipid metabolism of laying hens by increasing the abundance of Clostridia, Prevotellaceae, and Bifidobacteriaceae. Besides, Fan and Pedersen [39] reported that Proteobacteria and Fusobacteria were enriched in liver cirrhosis or nonalcoholic steatohepatitis. Contrarily, the abundances of Proteobacteria and Fusobacteria were decreased in HELP group compared to the CT group, and increased in FRT4L and FRT4H groups compared to the HELP group, while decreased in FRT4M group compared to the HELP group. However, the lipid contents in groups supplied with *Lp. plantarum* FRT4 were decreased. Thus, it is required to be further confirmed that how *Lp. plantarum* FRT4 improved the liver lipid metabolism in laying hens through altering Proteobacteria and Fusobacteria abundances in our future research. Although with a lower abundance, what the interesting was that *WPS*-2 was only identified in FRT4M group and identified as a biomarker. *WPS-2* is not culturable and its specific function has not yet been reported [40]. Zheng et al. [41] reported that *WPS-*2 was highly positively correlated with 4-hydroxyphenylpyruvic acid, which was related to L-tyrosine metabolism. However, it has not been reported that whether the *WPS*-2 played a role in regulating lipid metabolism in laying hens. This may provide a new idea to study the relationship between liver metabolism and gut microbiota.

The gut-liver axis theory considered that gut microbiota affected the metabolism of host, and the variation of liver metabolites could reflect the difference of gut microbiota composition [42]. In the present study, supplementation with *Lp. plantarum* FRT4 altered not only the composition of hepatic metabolites, but also cecal microbial structure. Besides, the results of the Spearman correlation analysis between gut microbiota and liver metabolites showed a greatly different relationship between HELP group compared to the CT group and FRT4M compared to the HELP group. Consequently, *Lp. plantarum* FRT4 could alleviate the HELP diet-induced FLHS through the “gut-liver” axis, and then heighten the laying performance of laying hens.

## Conclusions

Collectively, HELP diet resulted in lower laying performance, FLHS formation, and development in laying hens. Supplementation with *Lp. plantarum* FRT4 significantly increased laying performance, and attenuated FLHS with the decline of TG, TC, LDL-C, and the increase of HDL-C and VLDL-C in liver. Importantly, *Lp. plantarum* FRT4 regulated the different metabolites of PE and PC to affect the glycerophospholipid metabolic pathway. Meanwhile, *Lp. plantarum* FRT4 reshaped gut microbiota structure caused by HELP diet. In summary, *Lp. plantarum* FRT4 mitigated FLHS in laying hens through regulating liver lipid metabolism, liver function, and gut microbiota. In addition, the results showed that targeting glycerophospholipid metabolic pathway could be a potential and promising therapy method for FLHS in laying hens.

### Supplementary Information

**Additional file 1: Table S1.** Compositions and nutrients contents of experimental diets. **Table S2.** The primer sequences for qRT-PCR.

**Additional file 2: Fig. S1.** PCA analysis of evaluating the system stability through 7-fold cross validation (7 cycles of cross validation) for LC-MS. **Fig. S2.** Boxplot the metabolite strength of QC samples for LC-MS. **Fig. S3.** The plot of hierarchical clustering of metabolite expression for LC-MS. Supplementary **Fig.** **S4.** PCA analysis of evaluating the system stability through 7-fold cross validation (7 cycles of cross validation) for GC-MS. **Fig. S5.** Boxplot the metabolite strength of QC samples for GC-MS. **Fig. S6.** The plot of hierarchical clustering of metabolite expression for GC-MS.

**Additional file 3.** Deposited data.

**Additional file 4.** Deposited data.

## Data Availability

The data analyzed during the current study are available from the corresponding author on reasonable request. The datasets generated for this study can be found in NCBI https://dataview.ncbi.nlm.nih.gov/object/PRJNA1004760?reviewer=c9h5arl098kh2bqob1i1p1gmbd.

## Abbreviations

ACACA: Acetyl-CoA carboxylase alpha
ADFI: Average daily feed intake
ADMEC: Average daily metabolic energy consumption
CEPT1: Choline/ethanolamine phosphotransferase 1
CHKα: Choline kinase alpha
ETNK: Ethanolamine kinase
FABP1: Fatty acid binding protein 1
FASN: Fatty acid synthase
FLHS: Fatty liver hemorrhage syndrome
GC-MS: Gas chromatography-mass spectrometry
GDE1: Glycerophosphodiester phosphodiesterase 1
GDPD5: Glycerophosphodiester phosphodiesterase domain containing 5
HDL-C: High-density lipoprotein cholesterol
HELP: High-energy low-protein
LC-MS: Liquid chromatography-mass spectrometry
LDA: Linear discriminant analysis
LDL-C: Low-density lipoprotein cholesterol
LEfSe: Linear discriminant analysis effect size
*Lp. plantarum*: *Lactiplantibacillus plantarum*
LPCAT2: Lysophosphatidylcholine acyltransferase 2
LPCAT3: Lysophosphatidylcholine acyltransferase 3
MAFLD: Metabolic associated fatty liver disease
ME1: Malic enzyme 1
MTTP: Microsomal triglyceride transfer protein
NAFLD: Non-alcoholic fatty liver disease
OPLS-DA: Orthogonal Partial Least-Squares-Discriminant Analysis
PBS: Phosphate buffered saline
PC: Phosphatidylcholine
PCYT1α: Phosphate cytidylyltransferase 1 alpha
PCYT2: Phosphate cytidylyltransferase 2
PE: Phosphatidylethanolamine
PISD: Phosphatidylserine decarboxylase
PLA2G6: Phospholipase A2 group VI
SCD-1: Stearoyl-CoA desaturase-1
SREBP-1: Sterol regulatory element binding protein 1
TC: Total cholesterol
TG: Triglyceride
VIP: Variable importance of projection
VLDL-C: Very low-density lipoprotein cholesterol
VLDLR: Very low-density lipoprotein receptor
