# Supplementary material for: Lactiplantibacillus plantarum FRT4 attenuates high-energy low-protein diet-induced fatty liver hemorrhage syndrome in laying hens through regulating gut-liver axis
Source: J Anim Sci Biotechnol. 2024 Feb 21;15:31. doi: 10.1186/s40104-023-00982-6 (PMC10880217; doi:10.1186/s40104-023-00982-6)
Supplement: Supplementary file 1 — Additional file 1: Table S1. Compositions and nutrients contents of experimental diets. Table S2. The primer sequences for qRT-PCR. [file 40104_2023_982_MOESM1_ESM.docx]

**Additional file 1**

**Table S1** Compositions and nutrients contents of experimental diets

| **Items** | **Normal diet** | **HELP diet^1^** |
| --- | --- | --- |
| Ingredients, % |  |  |
| Corn | 62.50 | 64.50 |
| Soybean meal | 26.50 | 18.50 |
| Lard | - | 6.00 |
| Limestone | 9.00 | 9.00 |
| CaHPO_4_ | 0.90 | 0.90 |
| Salt | 0.30 | 0.30 |
| Choline chloride | 0.12 | 0.12 |
| DL-Methionine | 0.16 | 0.16 |
| Phytase | 0.02 | 0.02 |
| Premix^2^ | 0.50 | 0.50 |
| Total | 100 | 100 |
| Nutrient levels^3^ |  |  |
| GE^4^, MJ/kg | 17.66 | 19.69 |
| Crude protein^5^, % | 16.09 | 13.03 |
| ME, MJ/kg | 11.26 | 13.08 |
| Ca, % | 3.51 | 3.50 |
| Available P, % | 0.47 | 0.46 |
| Met, % | 0.37 | 0.35 |
| Lys, % | 0.78 | 0.76 |

^1^ HELP diet: High-energy low-protein diet

^2^ The premix provided the followings per kg of diet: vitamin A 11,000 IU, vitamin D 22,500 IU, vitamin E 80 IU, vitamin K 2 mg, vitamin B_12_ 0.02 mg, thiamine 1 mg, niacin 36 mg, biotin 0.5 mg, folic acid 0.5 mg, Mn 63 mg, Fe 60 mg, I 2 mg, Cu 8 mg, Zn 66 mg, Se 0.3 mg

^3^ Nutrient levels: Nutrient levels were calculated values

^4^ GE: Gross energy. Measured value

^5^ Crude protein: Measured value

**Table S2** The primer sequences for qRT-PCR

| **Gene name** | **Primer sequence (5´→3´)** | **Accession number** | **Product size, bp** |
| --- | --- | --- | --- |
| *β-actin* | TTGTTGACAATGGCTCCGGT  TCTGGGCTTCATCACCAACG | NM_205518.2 | 153 |
| *SREBP-1* | GAGACCATCTACAGCTCCGC  CATCCGAAAAGCACCCCTCT | NM_204126.3 | 154 |
| *FASN* | GCTAAGATGGCATTGCACGG  TCCATTCAGTTCCAGACGGC | NM_205155.4 | 135 |
| *SCD-1* | AGCAGAACGAGGCATGGTAG  GGATCAGCGTCAGCCCAATA | NM_204890.2 | 146 |
| *ACACA* | TTGTGGCACAGAAGAGGGAA  GTTGGCACATGGAATGGCAG | NM_205505.2 | 161 |
| *ME1* | CTCACCATGAAGAGGGGCTAC  AGCAAGGTGGTAACAGTCCAT | NM_204303.2 | 115 |
| *FABP1* | GGGAAGAGTGTGAGATGGAGC  GTTCGGTCACGGATTTCAGC | NM_204192.4 | 114 |
| *MTTP* | CCACGGTGCAGTTTTCACAG  TTCCCTCTCCTCGCAGTGTA | NM_001109784.3 | 133 |
| *LPL* | GTGAAGGATGGGAGGGACAGC  CCCTACAAATCAACCTGGCTCC | NM_205282.2 | 109 |
| *VLDLR* | TCTGAGATGTGGAGGATTCAAC  GCCGTCACCAGCAGTAAGAT | NM_205229.2 | 105 |
| *ETNK1* | AAGCTGGACATCACCGTCAG  CGTCCGTTGTATCACCCACA | XM_416426.8 | 172 |
| *ETNK2* | GATTGCTGGGAAACGGAACG  CGTCAGTGAAGAGCTTGGTC | XM_424313.8 | 178 |
| *PCYT2* | GCTCCTTATGTCACCACGCT  CAACGAGGTCGGTAGTGGAC | NM_001291966.1 | 175 |
| *CEPT1* | ACAAACAGCAGCACTCCACT  CATCCAGTCAGGGTTCGTCC | NM_001006392.3 | 120 |
| *GDE1* | ACAGCTTCTTGTGGCGGTTA  CTGTCCAGGCAACCACTTGA | XM_015294545.4 | 117 |
| *GDPD5* | CCAGAAGTGGCGCTTAGGAA  GTGACCATCTCGTTGGCGTA | NM_001305150.2 | 195 |
| *CHKα* | AAAGAGAGAGCTGCTTCCGTG  GCACCATATAAGCGCAGCAG | XM_046942358.1 | 123 |
| *PCYT1α* | CAGAAGGCGCACTGAAACAC  CTTGGTTGAAAAGGGCCAGC | XM_046898487.1 | 140 |
| *PLA2G6* | TACCGGGTGAAGGAGGTGTC  TCAAAGAGCACCAGGGCTTC | NM_001130738.2 | 185 |
| *LYPLA2* | GACCTGATGGGCTTGACTCC  GGATGATACGGTTGGGTGGG | NM_001162382.1 | 124 |
| *LPCAT2* | TCGTTCCCAATCCCTTCGTC  CCTCCAGTGTGACGGAAAGT | NM_001030568.3 | 166 |
| *LPCAT3* | CGCCTGAGTTTGGGTCTCTT  GTAACCACAGCGGAACCAGA | XM_416516.6 | 120 |
| *CHO1* | TCCTCTTCATGGTTGACCGC  GTGAGAACAGGACAGCCACA | XM_003642538.6 | 108 |
| *PISD* | CAGCCACCTTCCAGGACATT  TGTATCCTGCGTATCCCCCA | XM_004934365.5 | 124 |
| *PEMT* | CGGCCTGGATTACAGCGAG  TGCTCCCATCTTGCTACCAC | NM_001006164.2 | 144 |
