# Supplementary material for: Lactiplantibacillus plantarum FRT4 attenuates high-energy low-protein diet-induced fatty liver hemorrhage syndrome in laying hens through regulating gut-liver axis
Source: J Anim Sci Biotechnol. 2024 Feb 21;15:31. doi: 10.1186/s40104-023-00982-6 (PMC10880217; doi:10.1186/s40104-023-00982-6)
Supplement: Supplementary file 2 — Additional file 2: Fig. S1. PCA analysis of evaluating the system stability through 7-fold cross validation (7 cycles of cross validation) for LC-MS. Fig. S2. Boxplot the metabolite strength of QC samples for LC-MS. Fig. S3. The plot of hierarchical clustering of metabolite expression for LC-MS. Supplementary Fig. S4. PCA analysis of evaluating the system stability through 7-fold cross validation (7 cycles of cross validation) for GC-MS. Fig. S5. Boxplot the metabolite strength of QC samples for GC-MS. Fig. S6. The plot of hierarchical clustering of metabolite expression for GC-MS. [file 40104_2023_982_MOESM2_ESM.docx]

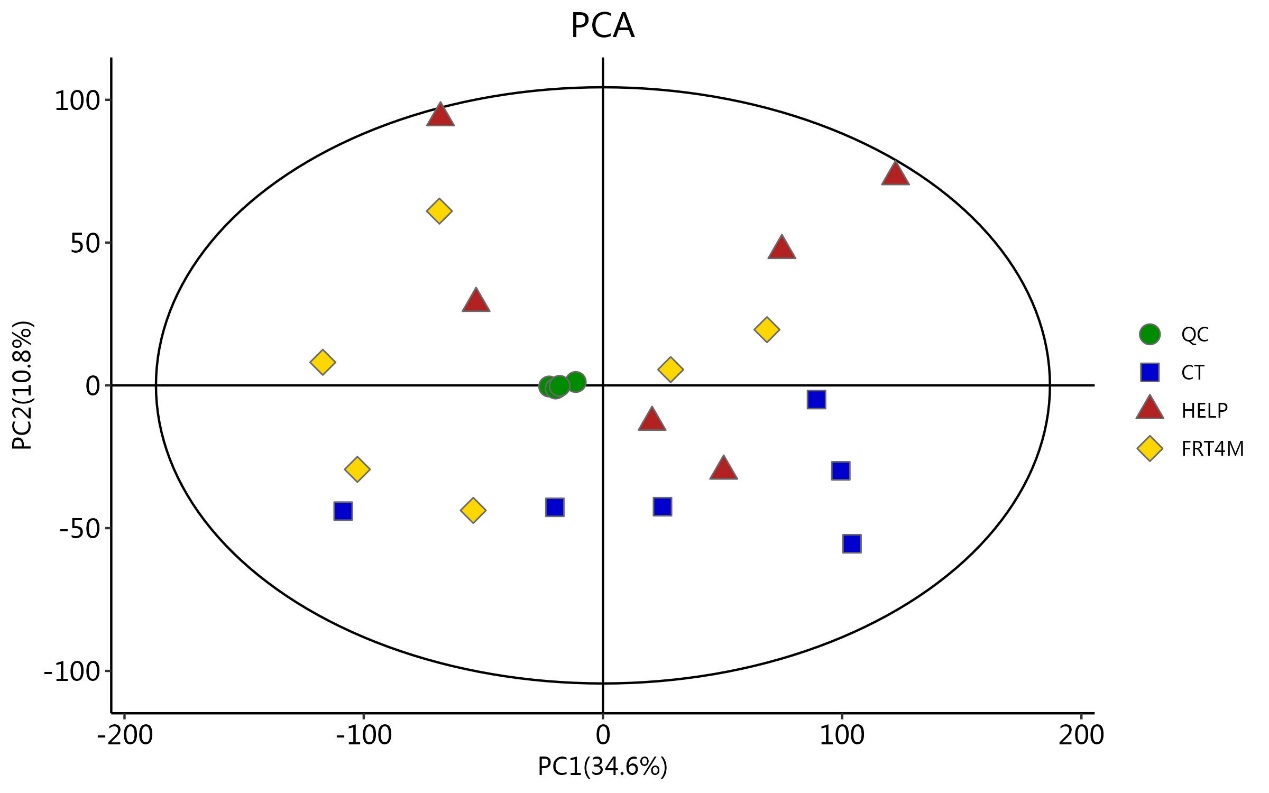


**Fig. S1** PCA analysis of evaluating the system stability through 7-fold cross validation (7 cycles of cross validation) for LC-MS. QC samples are closely clustered together, indicating that this experiment has good stability and repeatability. The abscissa PC1 is the first principal component interpretation rate, and the ordinate PC2 is the second principal component interpretation rate. Each point in the graph represents a sample. If the samples are clustered together, it indicates that the differences between these samples are small; conversely, the farther the distance between samples, the greater the difference between samples


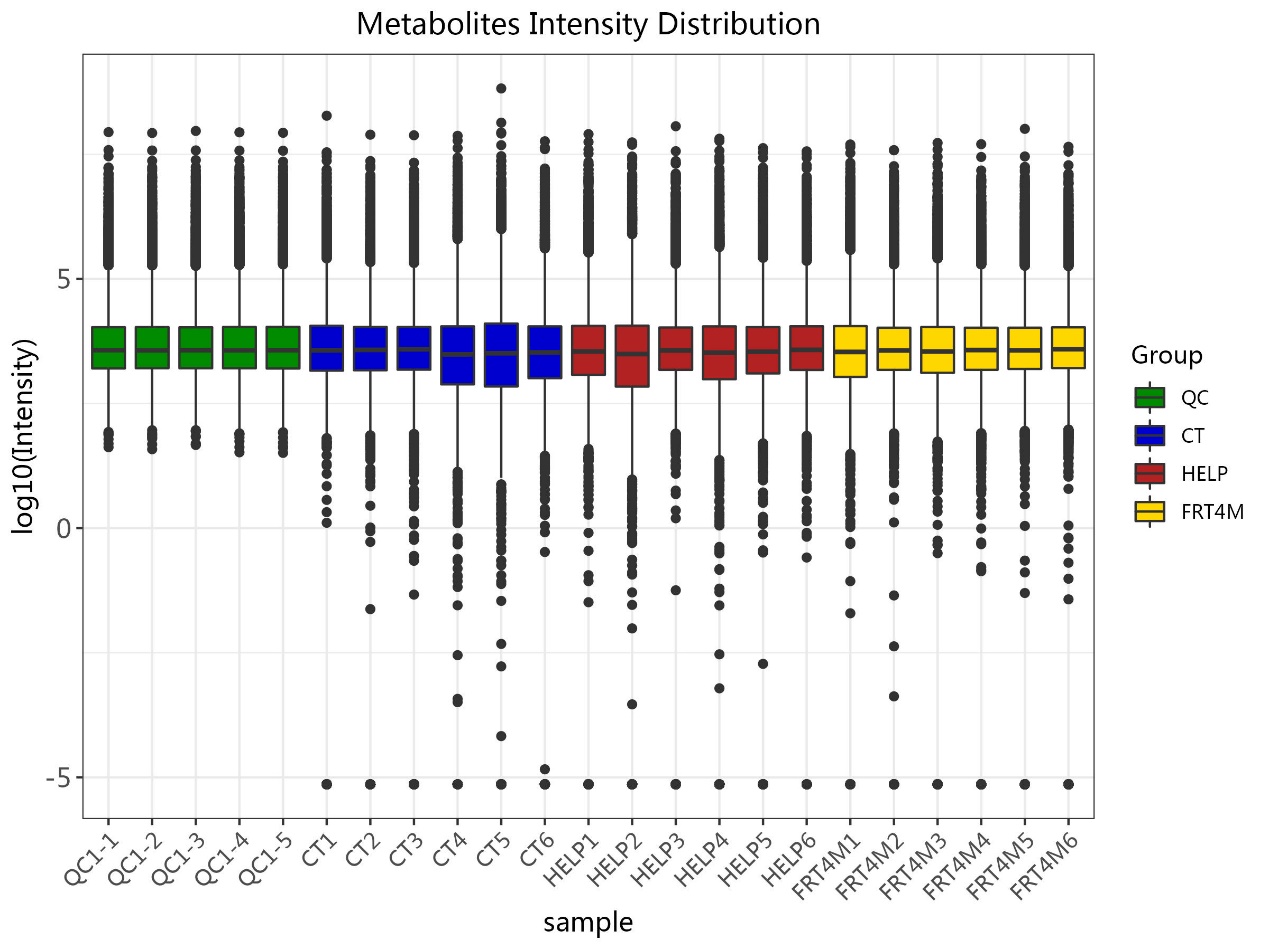
**Fig. S2** Boxplot the metabolite strength of QC samples for LC-MS. The abscissa is the sample number, and the ordinate is the log10 value of each metabolite abundance in the sample. It mainly reflects the characteristics of the distribution of metabolite abundance values in each sample, and is used to evaluate QC consistency and intragroup repeatability


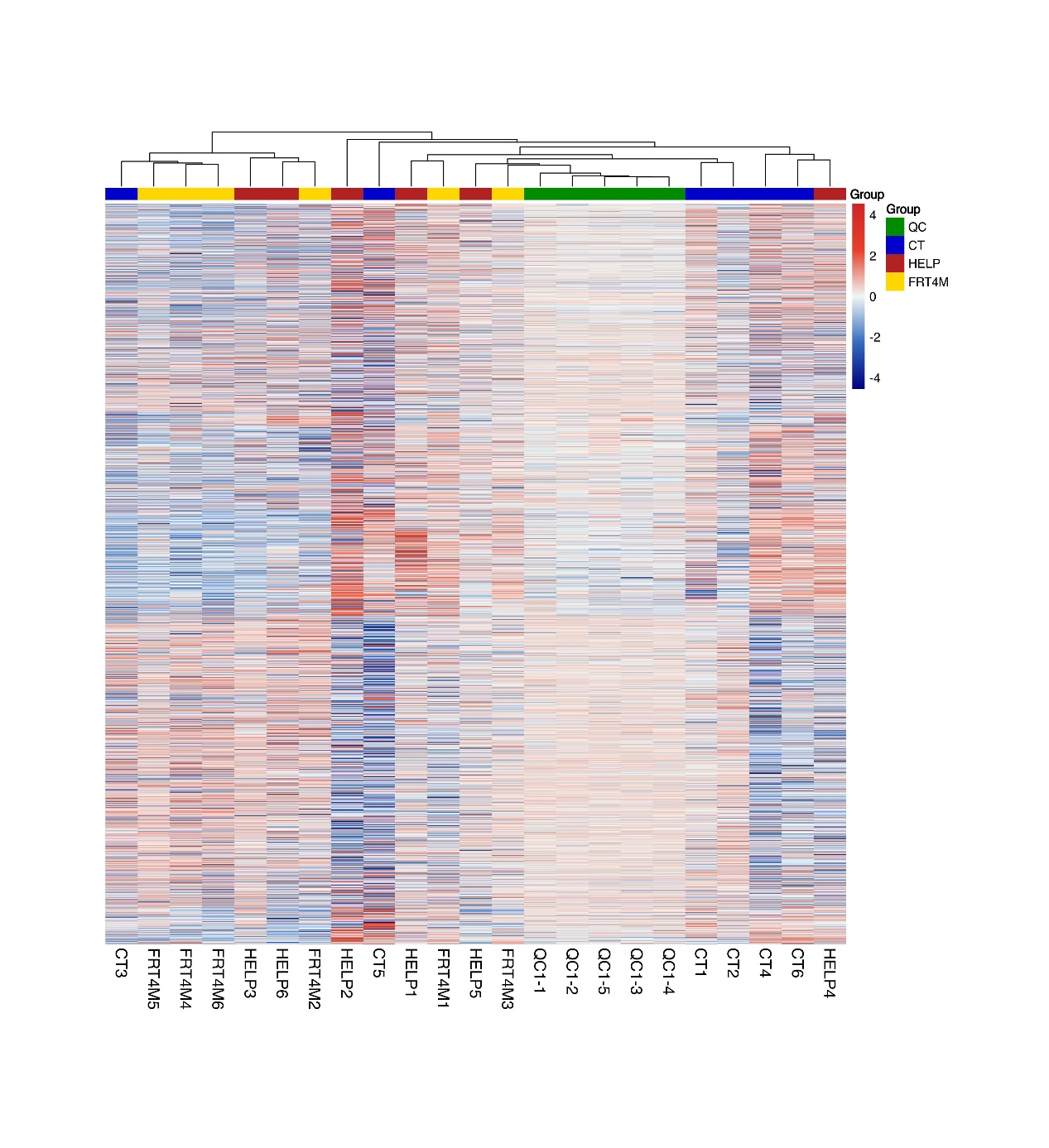


**Fig. S3** The plot of hierarchical clustering of metabolite expression for LC-MS. To more intuitively display the relationship between QC samples and other samples and the stability between QC samples. Each column is a sample, and each row is a metabolite. The relative abundance of metabolites in the color body will be clustered together if the samples are similar. It is mainly used to evaluate QC consistency, intra group repeatability, and inter sample similarity


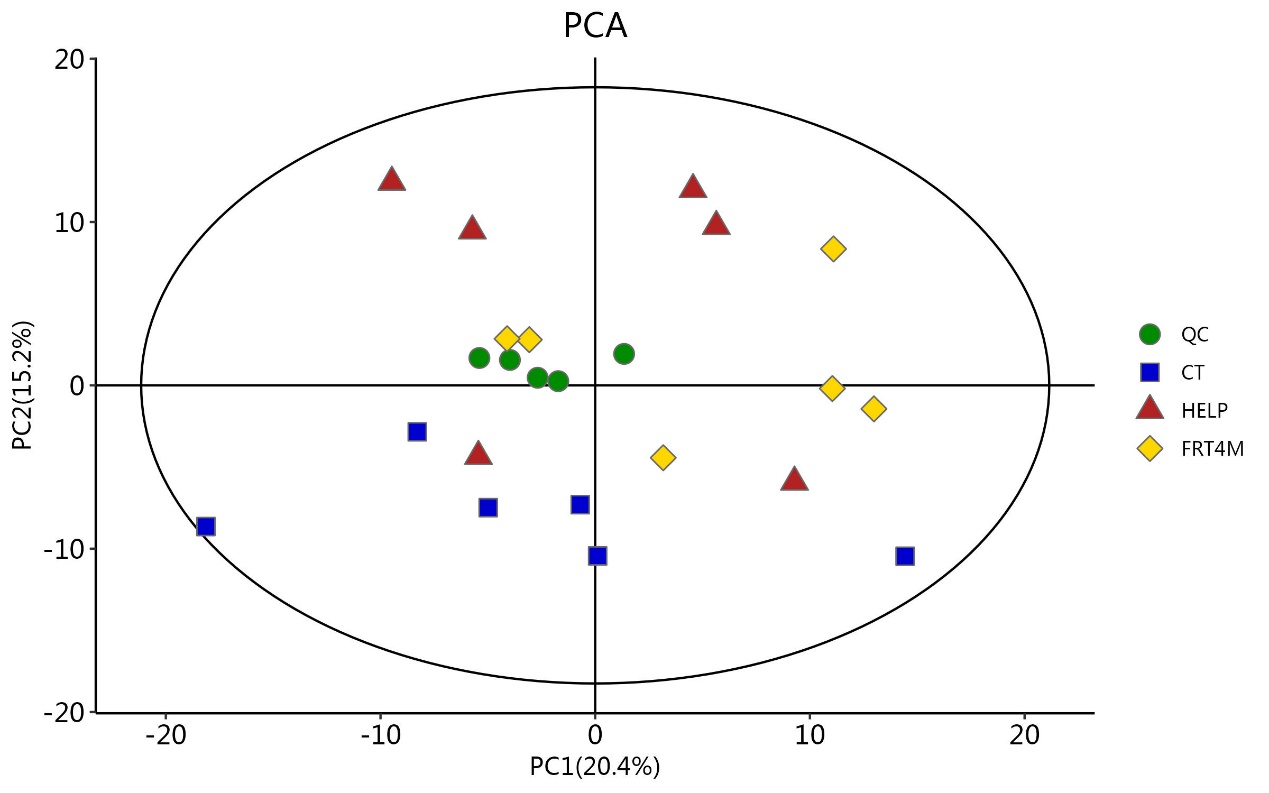


**Fig. S4** PCA analysis of evaluating the system stability through 7-fold cross validation (7 cycles of cross validation) for GC-MS. QC samples are closely clustered together, indicating that this experiment has good stability and repeatability. The abscissa PC1 is the first principal component interpretation rate, and the ordinate PC2 is the second principal component interpretation rate. Each point in the graph represents a sample. If the samples are clustered together, it indicates that the differences between these samples are small; conversely, the farther the distance between samples, the greater the difference between samples


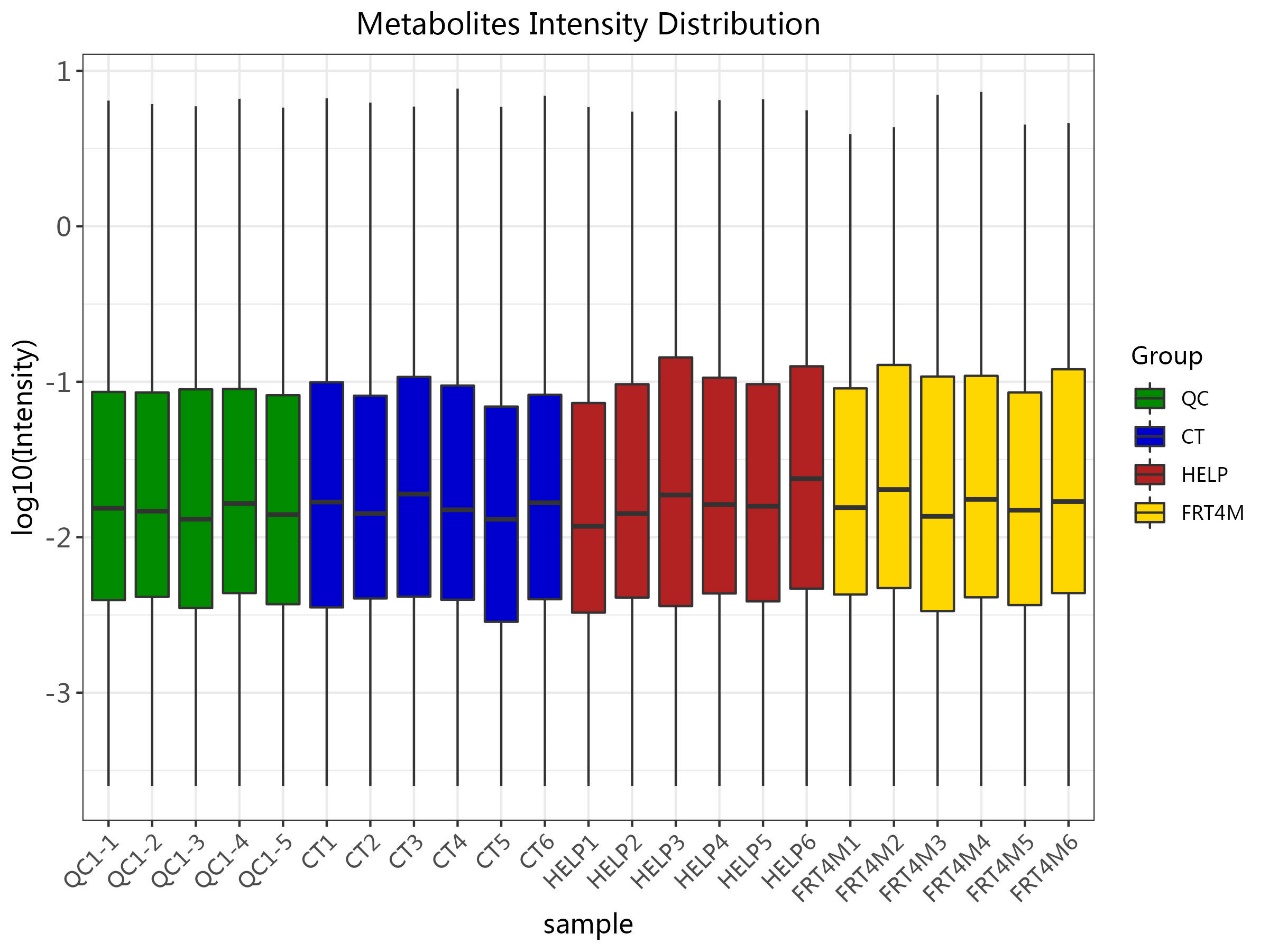


**Fig. S5** Boxplot the metabolite strength of QC samples for GC-MS. The abscissa is the sample number, and the ordinate is the log10 value of each metabolite abundance in the sample. It mainly reflects the characteristics of the distribution of metabolite abundance values in each sample, and is used to evaluate QC consistency and intragroup repeatability


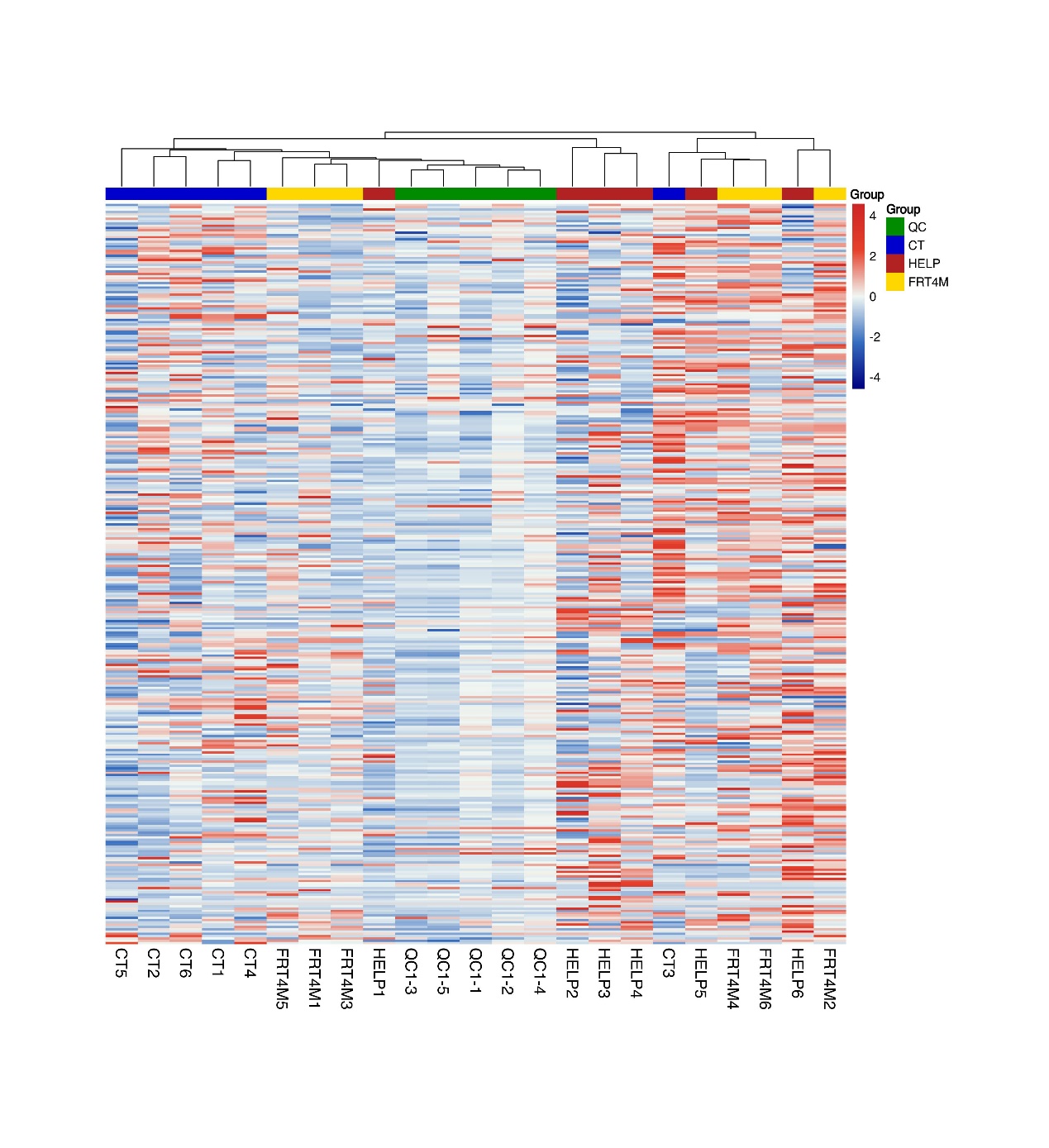


**Fig. S6** The plot of hierarchical clustering of metabolite expression for GC-MS. To more intuitively display the relationship between QC samples and other samples and the stability between QC samples. Each column is a sample, and each row is a metabolite. The relative abundance of metabolites in the color body will be clustered together if the samples are similar. It is mainly used to evaluate QC consistency, intra group repeatability, and inter sample similarity
